# Recent paclitaxel formulation strategies: expanding the therapeutic index by addressing biopharmaceutical and toxicity limitations

**DOI:** 10.1007/s12272-026-01609-w

**Published:** 2026-04-10

**Authors:** Jihoon Lee, Min-Koo Choi, Im-Sook Song

**Affiliations:** 1 College of Pharmacy and Research Institute of Pharmaceutical Sciences, Kyungpook National University, 80 Daehakro, Daegu, 41566 Republic of Korea; 2 BK21 FOUR Community‑Based Intelligent Novel Drug Discovery Education Unit and Vessel‑Organ Interaction Research Center (VOICE), Kyungpook National University, Daegu, 41566 Republic of Korea; 3 College of Pharmacy, Dankook University, Cheonan, 31116 Republic of Korea

**Keywords:** Paclitaxel (PTX), Dose limiting toxicities, Formulation, Administration route

## Abstract

Paclitaxel (PTX) is a standard-of-care antineoplastic agent that stabilizes microtubules. However, its clinical utility is limited by dose limiting toxicities (e.g., myelosuppression and neuropathy), influencing decades of formulation development. Therefore, this review aims to analyze formulation strategies developed to address the three primary limitations. First, solvent-free formulations (e.g., albumin-bound PTX) mitigated the solvent-related toxicity and nonlinear pharmacokinetics of Cremophor EL-based PTX, improving the safety profile by eliminating hypersensitivity reactions. Second, intravenous formulations using liposomes or polymeric micelles for mitigating dose limiting toxicities via prolonged circulation and enhanced tumor retention demonstrated inconsistent clinical translation. Recently, active targeting platforms aimed to keep PTX largely inactive systemically while promoting mechanism-triggered delivery or tumoral uptake are under preclinical evaluation to expand the therapeutic index. Third, poor oral bioavailability, attributed to P-glycoprotein (P-gp)-mediated efflux and first-pass metabolism, was addressed via two strategies: gut-specific P-gp inhibition and lipid-based bypass formulations; however, interpatient absorption variability remains limited. In this review, decades of formulation research, tracing the evolution of PTX from a challenging molecule to a versatile therapeutic platform, were synthesized, highlighting the effect of addressing key biopharmaceutical and toxicity limitations in expanding its therapeutic index. Additionally, we examined the factors contributing to the frequent failure of passive targeting strategies to separate tumor exposure from systemic toxicity in human solid tumors. In conclusion, understanding PTX formulation strategies may facilitate future therapies to adopt flexible administration routes, incorporate biomarker-guided decision-making, and achieve controlled systemic exposure to reduce intrinsic dose-limiting toxicities.

## Introduction

Microtubules, essential cytoskeletal components, are present in all eukaryotic cells. They assemble into the mitotic spindle, mediating segregation of replicated chromosomes during cell division (Wordeman et al. 2021). Mitotic spindle assembly and function depend on microtubule dynamic instability, defined by stochastic switching between phases of slow polymerization and rapid depolymerization, even in the absence of external regulatory factors (Janson et al. 2003). Paclitaxel (PTX) promotes tubulin polymerization and stabilizes microtubules, thereby suppressing depolymerization. Consequently, microtubules lose their dynamic instability, leading to rigid spindle structures that fail to properly segregate chromosomes (Horwitz 1994).

Given that PTX targets microtubules, it is effective across several solid tumors and is a core agent in modern chemotherapy. The GOG-111 trial shows that PTX/cisplatin is superior to the then-standard regimen of cisplatin/cyclophosphamide as first-line treatment for advanced ovarian cancer. The PTX combination arm significantly improves the overall response rate (ORR; 73% vs. 60%) and overall survival (OS; 38 vs. 24 months) (McGuire et al. 1996). The European-Canadian OV-10 study confirms these findings, establishing the PTX–platinum regimen as the standard first-line therapy for advanced ovarian cancer (Marchetti et al. 2014).

PTX is crucial for treating adjuvant and metastatic breast cancer (Khanna et al. 2015). In the CALGB 9342 study, PTX dose was set at 175 mg/m^2^ as the optimal dose in patients with metastatic breast cancer, showing no additional clinical benefit at higher doses (210 ~ 250 mg/m^2^) (Harris et al. 2006; Lichtman et al. 2012). Additionally, PTX combined with a platinum agent (most commonly carboplatin) constitutes a backbone regimen for first-line treatment of advanced non–small cell lung cancer (NSCLC) (Khanna et al. 2015). In a phase III trial, the Abraxane (albumin-bound PTX formulation)/gemcitabine combination significantly improves OS (8.5 vs. 6.7 months) and ORR (23% vs. 7%) compared to gemcitabine monotherapy, establishing this regimen as a first-line standard of care for metastatic pancreatic adenocarcinoma (Von Hoff et al. 2013). In metastatic melanoma studies, PTX is evaluated both as monotherapy and in combination with other agents. Although chemotherapy responses in melanoma are typically limited, PTX shows clinical activity in selected patient populations (Hauschild et al. 2009). Several phase II trials evaluating PTX combined with carboplatin report ORRs of approximately 11–22%, with minimal effect on OS in patients with advanced melanoma (O’Day et al. 2009). Consequently, the carboplatin–PTX combination, characterized by lower toxicity and greater ease of administration, is a widely used standard therapy globally (Schiller et al. 2002).

Despite extensive clinical validation, PTX faces several challenges that require optimization, including formulation-related toxicity, limitations in administration routes, and persistent dose-limiting toxicities (DLTs), such as myelosuppression and peripheral neuropathy (Jackson et al. 2021). To evaluate newly developed PTX formulations in the safety concern, an exposure-based framework should be used. In this context, the dose ratio between the maximum tolerated dose (MTD) and the effective dose defines the therapeutic index (Colombo and Rich 2022; Chen et al. 2024). This index provides a quantitative measure of the relative safety of a drug in both clinical and preclinical stages. Therefore, this review aims to analyze recent formulation strategies in terms of the mechanistic pharmacology, biopharmaceutical limitations, and toxicity concerns using the therapeutic index framework.

## Mechanisms of action and toxicity of paclitaxel

### Molecular mechanism of paclitaxel

PTX binds to the β-tubulin taxane-binding pocket on the luminal surface of microtubules, stabilizing the flexible M-loop and reinforcing the microtubule lattice (Prota et al. 2023). Consequently, PTX suppresses microtubule dynamic instability, reducing depolymerization frequency and enhancing polymerization, leading to an accumulation of stabilized microtubules (Smith et al. 2021). Prolonged mitotic arrest induces cellular stress and activates the intrinsic apoptotic pathway, promotes phosphorylation of Bcl-2 and Bax, and increases mitochondrial outer membrane permeabilization. Subsequent mitochondrial cytochrome c release activates a signaling cascade that activates caspase-3, resulting in apoptotic cell death (Wang et al. 2000).

**Fig. 1:**
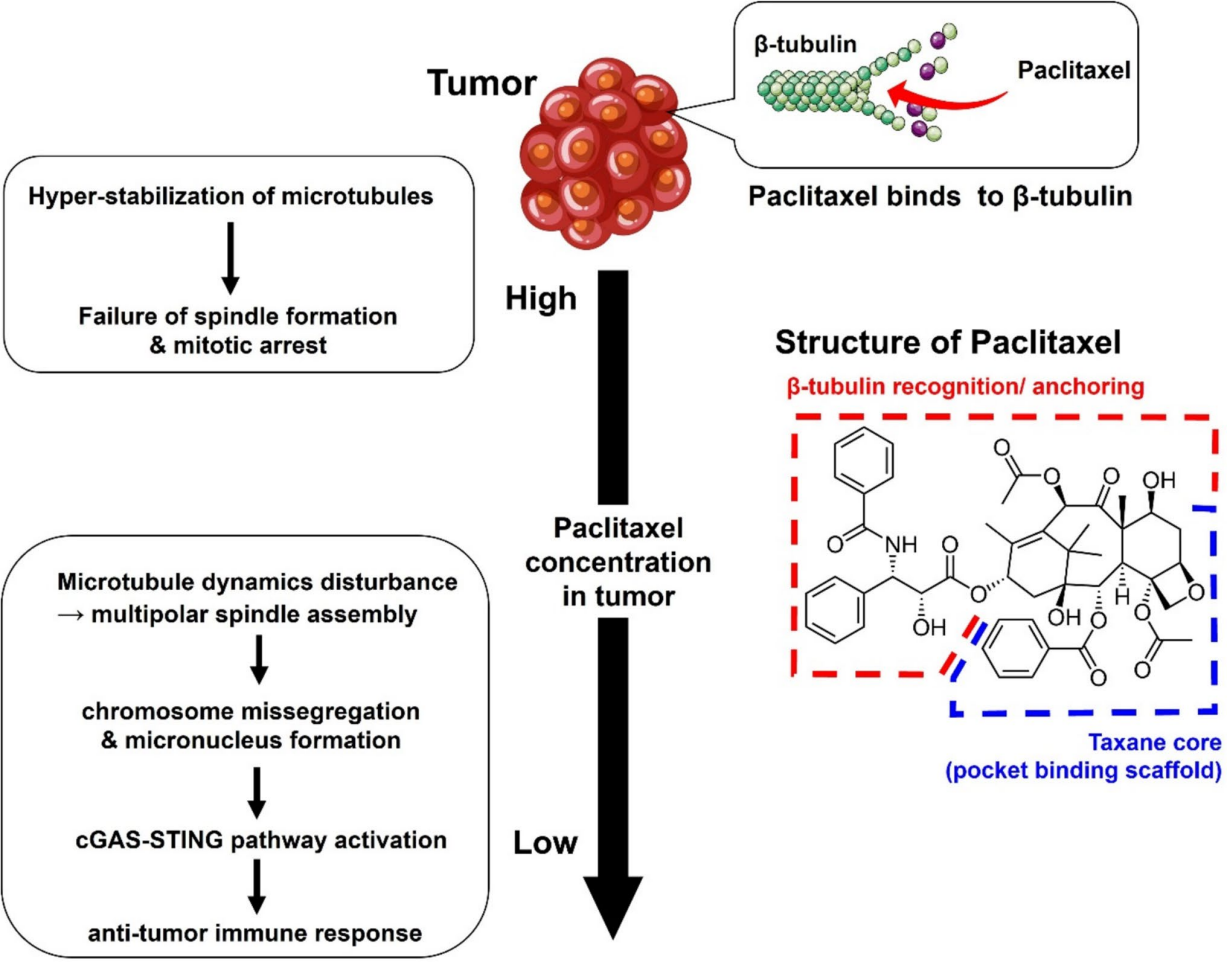
Concentration-dependent mechanisms of paclitaxel. At high concentrations (>1 μM), paclitaxel induces microtubule hyperstabilization and mitotic arrest. At clinically relevant concentrations (5–50 nM), it induces chromosomal mis-segregation and micronucleus formation, advancing the cGAS–STING pathway and stimulating an anti-tumor immune response. cGAS, cyclic GMP-AMP synthase; STING, stimulator of interferon genes. Paclitaxel chemical structure. Blue color indicatesβ-tubulin recognition/anchoring site and red color indicates pocket binding scaffold

The cytotoxic effects of PTX are concentration-dependent (Fig. 1). At micromolar PTX concentration (> 1 μM), PTX hyperstabilizes microtubules, induces mitotic arrest, and activates the canonical apoptotic pathway (Weaver et al. 2014). However, intratumoral PTX concentrations achieved in patients are typically insufficient to sustain complete mitotic arrest. At clinically relevant low nanomolar levels (5–50 nM), PTX disrupts microtubule dynamics and causes spindle pole fragmentation, thereby promoting multipolar spindle assembly and mitotic catastrophe. The resulting genomic instability ultimately leads to cell death (Weaver et al. 2014; Zasadil et al. 2014; Scribano et al. 2021). Recent studies show a key immunologic mechanism downstream of PTX-induced chromosome missegregation: lagging chromosomes often form micronuclei, and micronuclear envelope rupture exposes DNA to the cytosol, activating the cyclic GMP-AMP synthase stimulator of interferon genes (cGAS–STING) pathway (Lohard et al. 2020; Hu et al. 2021). Activating this pathway induces type I interferon signaling and proinflammatory gene transcription, thereby contributing to the therapeutic efficacy of PTX. Upon binding exposed double-stranded DNA in the cytosol, cytosolic cGAS catalyzes 2′3′-cyclic guanosine monophosphate–adenosine monophosphate (cGAMP). The resulting cGAMP binds STING on the endoplasmic reticulum, promoting its translocation to the Golgi apparatus and recruitment of TANK-binding kinase 1 and interferon regulatory factor 3 (IRF3). These signaling events activate type I interferon and nuclear factor kappa B (NF-κB)-mediated proinflammatory transcription. IRF3 and NF-κB pathway activation induce the expression of various microenvironment-modulating cytokines and chemokines (Bai et al. 2019; Zhang et al. 2025a, b). This interferon and chemokine signaling cascade activates dendritic cells and enhances CD8⁺ T-cell priming, thereby converting immunologically inactive tumors into immunologically active ones (Shen et al. 2025). Additionally, chromatin bridges attributed to defective mitosis directly activate cGAS, complementing the micronucleus-mediated mechanism (Serpico et al. 2023).

### Mechanisms of acquired resistance to paclitaxel and potential counter-strategy

Acquired resistance remains a major limitation of PTX therapy. Table 1 summarizes the primary mechanisms underlying this resistance and identifies targeted strategies for clinical circumvention. One key mechanism involves alterations in tubulin. Particularly, βIII-tubulin overexpression alters PTX binding and microtubule dynamics (Kanakkanthara et al. 2021). Clinical evidence supports this association: in various cancers, including NSCLC and ovarian cancer, lower βIII-tubulin expression correlates with higher response rates and longer survival among patients receiving PTX-based chemotherapy (Roque et al. 2013; Kyprianou et al. 2014). Beyond altered isotype expression, tubulin gene mutations alter the PTX-binding site and disrupt drug binding (Kanakkanthara et al. 2021). Furthermore, resistance involves additional mechanisms, including dysregulation of microtubule-associated proteins and the mechanical properties of the tumor microenvironment, warranting further investigation (Cui et al. 2020).

**Table 1:** Molecular mechanisms of tumor paclitaxel resistance

Mechanism	Molecular description	Potential counter-strategy
βIII-tubulin overexpression	Alters microtubule dynamics and reduces paclitaxel binding affinity	Select patients with low βIII-tubulin expression or use drugs binding alternative microtubule sites
Tubulin gene mutations	Induce structural changes at the paclitaxel-binding site that impair drug interaction	Use microtubule-targeting agents unaffected by the mutation or employ drugs with non-tubulin mechanisms
P-gp overexpression	Expels intracellular paclitaxel through ATP-dependent efflux, lowering active drug levels	Combine with P-gp inhibitors or use non-P-gp substrate drugs (e.g., cabazitaxel, ixabepilone)
Activation of PI3K/Akt or NF-κB pathways	Enhances pro-survival signaling that bypasses paclitaxel-induced apoptosis	Use pathway-targeted inhibitors (e.g., PI3K inhibitors) in combination with paclitaxel

*P-gp* P-glycoprotein, *NF-κB* nuclear factor kappa-light-chain-enhancer of activated B cells, *PI3K* phosphoinositide 3-kinase

In case of acquired resistance related to the alterations in tubulin such as βIII-tubulin overexpression or tubulin gene mutations, patients use alternative microtubule-targeting drugs. Eribulin and ixabepilone are particularly valuable where first-generation drugs fail, such as in tumors with βIII-tubulin alterations (Vrignaud et al. 2014). Eribulin, a synthetic analog of the sea sponge-derived halichondrin B, non-competitively binds to the plus (+) ends of microtubules to selectively suppress growth without affecting shortening (Seshadri et al. 2021). It is FDA approved for metastatic breast carcinoma refractory or relapsed on at least two prior treatment protocols for late-stage disease, including both anthracycline- and taxane-based chemotherapies (Trendowski 2015). In tumors exhibiting high βIII-tubulin expression, ixabepilone is being reconsidered as a treatment option for patients unlikely to benefit from further taxane therapy (Dumontet et al. 2009; Rau et al. 2020). In the Phase 3 EMERALD trial, eribulin combined with trastuzumab/pertuzumab was non-inferior to a PTX-based regimen as first-line therapy for human epidermal growth factor receptor 2 (HER2)-positive metastatic breast cancer, showing comparable PFS (14.0 vs. 12.9 months) (Yamashita et al. 2025). Eribulin significantly extended OS compared to treatment of physician’s choice (13.1 months vs. 10.6 months) in breast cancer patients pretreated with two to five prior chemotherapy regimens including an anthracycline and a PTX (Eslamian et al. 2017). Pooled analyses of phase III trials and real-world multicenter cohorts demonstrate that ixabepilone-based regimens retain clinically relevant activity in heavily pretreated metastatic or triple-negative breast cancer (Rugo et al. 2018; Gündüz et al. 2021).

Ixabepilone, an epothilone-class drug, binds to the same taxane pocket as PTX but it shows a clinical advantage to overcome taxane resistance. It also evades P-glycoprotein (P-gp) efflux pump and remains active in tumor cells overexpressing βIII-tubulin (Dumontet et al. 2009). Its clinical utility has been demonstrated in poor-prognosis subgroups, including patients with triple-negative breast cancer or early relapse (Cui et al. 2020). The combination of ixabepilone and capecitabine significantly increased the ORR (35% vs. 14%) compared to capecitabine alone in patients pre-treated with or resistant to taxanes (Sparano et al. 2010).

Another major resistance mechanism is enhanced PTX efflux from cancer cells, preventing the drug from achieving effective intracellular concentrations at its target. This process is primarily mediated by P-gp overexpression (Alalawy 2024). P-gp functions as an ATP-dependent efflux transporter actively exporting PTX and other chemotherapeutic agents, thereby reducing their intracellular concentrations to subtherapeutic levels. Additionally, P-gp upregulation activates downstream pro-survival signaling pathways, including PI3K/Akt and NF-κB, creating an integrated defense that combines intracellular anti-apoptotic signaling with enhanced drug efflux (Kang et al. 2011; Sui et al. 2021). The PI3K/Akt pathway upregulates anti-apoptotic factors, thereby increasing the apoptotic threshold. This pathway often cooperates with NF-κB signaling, which it activates to induce the transcription of additional survival-related genes, enabling cells to bypass PTX-induced apoptosis (Rascio et al. 2021).

In case of acquired resistance related to the overexpression of P-gp, patients use non-P-gp substratge drugs such as cabazitaxel, ixabepilone. Cabazitaxel is a semi-synthetic derivative of a natural taxoid, specifically engineered to maintain high potency in tumors that have developed resistance to earlier taxanes since it showed low affinity for P-gp (Sun et al. 2023). Cabazitaxel plus prednisone significantly extended mOS compared to mitoxantrone (15.1 vs. 12.7 months) in patients with metastatic castration-resistant prostate cancer previously treated with docetaxel (de Bono et al. 2010). While primarily approved for prostate cancer, Phase II studies in metastatic breast cancer have shown that cabazitaxel achieves an ORR of approximately 14% to 20% in patients who were confirmed to be resistant to PTX and docetaxel (Villanueva et al. 2011).

### Paclitaxel-associated toxicities

The potent antitumor activity of PTX is inherently associated with toxicity. Myelosuppression is the most common DLT, with neutropenia as its primary manifestation. PTX-induced neutropenia is attributed to inhibition of proliferation in bone marrow progenitor cells, leading to decreased circulating neutrophil counts. This toxicity is influenced by dosing schedule and systemic exposure; time above threshold concentration (e.g., T_c_ > 0.05 μM) is among the most robust predictors of PTX-associated neutropenia (Muth et al. 2021). Therefore, PTX therapy using novel delivery platforms should be evaluated based on their ability to regulate exposure of pharmacologically active PTX—particularly by minimizing peak concentrations and optimizing T_c_ (Henningsson et al. 2001).

Peripheral neuropathy is another major DLT that correlates with cumulative PTX exposure. PTX-induced peripheral neuropathy is attributed to microtubule stabilization in neurons, disrupting axonal transport and promoting nerve degeneration. Mitochondrial dysfunction and neuroinflammation further contribute to toxicity, making the dorsal root ganglia (DRG) and long peripheral nerves particularly susceptible (Klein and Lehmann 2021). Clinically, it predominantly presents as sensory symptoms in a stocking-and-glove distribution in the hands and feet (e.g., numbness, pain, burning sensations) (Nakashima et al. 2012). The incidence and severity of peripheral neuropathy correlate with cumulative PTX exposure and T_c_ (Hertz et al. 2018; Stage et al. 2018). Duloxetine is the only evidence-based therapy for managing painful neuropathy. Additional strategies include PTX dose adjustment (Loprinzi et al. 2020). PTX-induced peripheral neuropathy results from axonal damage. Axonal injury induces the release of neurofilament light chain (NfL), a neuronal structural protein, into the bloodstream. Recent studies show that serum NfL (sNfL) levels increase during PTX treatment and correlate strongly with neuropathy severity (Mortensen et al. 2023). An early increase in sNfL (e.g., at week 3) may predict subsequent development of severe neuropathy (Karteri et al. 2022). In prospective cohorts of patients receiving PTX-based chemotherapy for gynecologic and breast cancers, serial sNfL measurements closely track both the onset and severity of peripheral neuropathy. On-treatment increases in sNfL predict subsequent clinically significant neurotoxicity, supporting its potential as an objective biomarker for early detection and risk-adapted dose adjustments (Karteri et al. 2022; Kim et al. 2022).

### Toxicities of cremophor EL-based paclitaxel formulations

Cremophor EL (CrEL), a polyoxyethylated castor oil-based surfactant, facilitates the development of intravenous (IV) PTX formulation. CrEL solubilizes PTX in micelles, preventing precipitation and crystallization (Lu et al. 2013). However, its most significant adverse effect is anaphylactoid hypersensitivity, ranging from mild pruritus to life-threatening systemic reactions, occurring in approximately 41% of patients (Gallego-Jara et al. 2020). CrEL exposure is also associated with altered plasma lipid profiles, hyperlipidemia, erythrocyte aggregation, and development of peripheral neuropathy (Scripture et al. 2005; Kim et al. 2016).

**Fig. 2:**
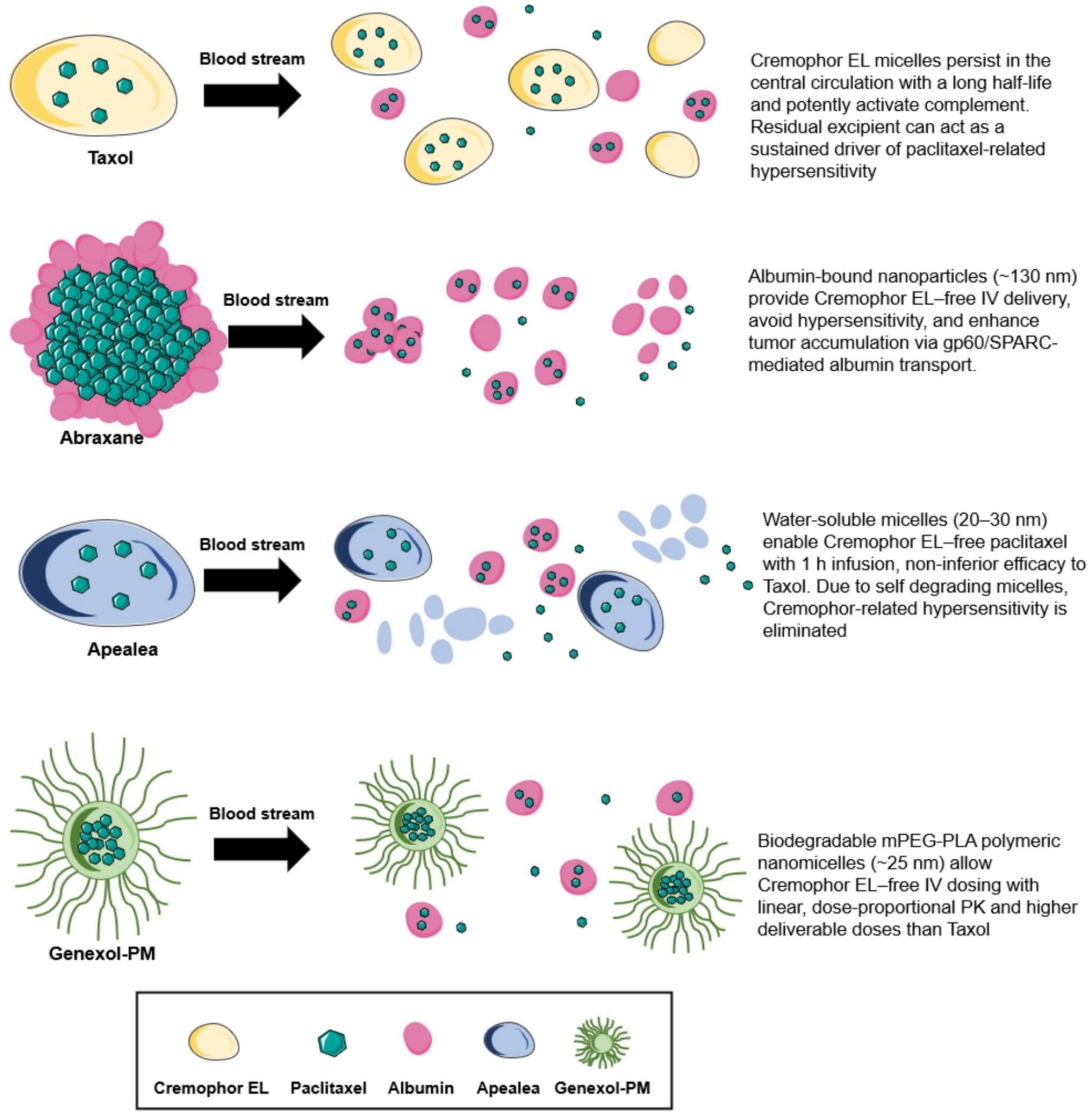
Comparative plasma behavior of paclitaxel formulations. Schematic representation showing the distinct release and carrier dynamics of Taxol, Abraxane, Apealea, and Genexol-PM. CrEL micelles sequester paclitaxel and contribute to neurotoxicity, while Abraxane rapidly distributes via albumin-mediated transport. Apealea forms self-degrading micelles that release paclitaxel quickly, while Genexol-PM provides controlled release through gradual dissociation of biodegradable polymeric micelles

Clinical pharmacokinetic studies show that CrEL is eliminated slowly in humans, with a terminal half-life of approximately 84 h, limited distribution beyond the plasma compartment, and measurable plasma levels persisting 1 week after a single PTX infusion (Sparreboom et al. 1998). PTX formulated with CrEL and ethanol, marketed as Taxol, strongly activates the complement cascade in human serum, serving as the primary mechanism underlying CrEL-mediated toxicities. Administering CrEL-based formulations in large infusion volumes destabilizes micelles, leading to PTX reprecipitation. Free CrEL released from disrupted micelles may directly contribute to hypersensitivity reactions via complement activation and histamine release (Szebeni 2005; El Nemr et al. 2018). Since complement activation-related pseudoallergy is a well-recognized mechanism of acute infusion reactions to micellar and surfactant-based nanomedicines, the prolonged systemic exposure to complement-activating CrEL provides a plausible explanation for the hypersensitivity profile of conventional PTX formulations (Fülöp et al. 2018) (Fig. 2). Consequently, CrEL-based formulations require routine premedication (e.g., steroids and antihistamines), while CrEL-free formulations are increasingly preferred.

## Pharmacokinetic properties of paclitaxel governing the efficacy and toxicity

PTX is a hydrophobic, white crystalline compound with low aqueous solubility (0.7–2 μg/mL), which limits its clinical application (Gallego-Jara et al. 2020). To address this limitation, PTX was formulated in a 1:1 mixture of CrEL and ethanol, serving as a surfactant-based solvent system enabling IV administration. The U.S. Food and Drug Administration (FDA) and the European Medicines Agency (EMA) have approved Taxol for the treatment of breast, ovarian, and lung cancers, along with Kaposi sarcoma (Table 2). In this Chapter, beginning with Taxol, the formulation characteristics, pharmacokinetic properties, clinical outcomes, and toxicity profiles of currently approved PTX formulations are discussed.

**Table 2:** Clinically approved paclitaxel formulations used in the clinic: approval year, region, route, indications, and related companies

Product (formulation type)	Approval year	Nation (Agency)	Route	Target tumor (approved / widely used examples)	Company
Taxol (solvent-based/mixture of Cremophor EL/EtOH)	1992 1993	USA (FDA) EU (EMA)	IV infusion	Ovarian cancer (initial) later expanded to breast, NSCLC, Kaposi’s sarcoma etc	Bristol-Myers Squibb
Abraxane® (nab-paclitaxel; albumin-bound nanoparticle)	2005 2008	USA (FDA) EU (EMA)	IV infusion	Metastatic breast cancer (initial) later NSCLC (with carboplatin), pancreatic (with gemcitabine) etc	American Biosciences/Abraxis → Celgene/BMS
Lipusu (liposomal paclitaxel)	2003	China (NMPA)	IV infusion	Approved indication (for ovarian, breast, NSCLC)	Luye Pharma Group
Genexol-PM (polymeric micelle)	2006	Republic of Korea (MFDS)	IV infusion	Metastatic/recurrent breast cancer NSCLC (reported Korean approval/clinical use)	Samyang Biopharm
Nanoxel (polymeric micelle nanoparticle)	2006	India (DCGI)	IV infusion	Metastatic breast cancer (DCGI approval reported) broader clinical use reported	Dabur Pharma
Apealea (paclitaxel micelle)	2018	EU (EMA)	IV infusion	Ovarian / fallopian tube / primary peritoneal cancer (with carboplatin)	Oasmia/Vivesto Inceptua. Currently, no longer authorized in EU
Liporaxel (SMEDDS)	2016	Republic of Korea (MFDS)	Oral	Gastric cancer (Korea approval reported)	Daehwa Pharmaceutical

*CrEL* Cremophor EL, *EtOH* ethanol, *IV* intravenous, nab-paclitaxel, nanoparticle albumin-bound paclitaxel, *FDA* U.S. Food and Drug Administration, *EMA* European Medicines Agency, *NMPA* National Medical Products Administration (China), *MFDS* Ministry of Food and Drug Safety, *DCGI* Drugs Controller General of India, *NSCLC* non-small cell lung cancer, *SMEDDS* self-emulsifying drug delivery system

### Nonlinear pharmacokinetics of cremophor EL-based paclitaxel formulations

The use of CrEL as a surfactant results in sequestration of PTX within CrEL micelles. Consequently, most circulating PTX remains micelle-associated, and only a small fraction (< 1–2%) is unbound and pharmacologically active. This micellar sequestration leads to significant nonlinear pharmacokinetics (Brouwer et al. 2000; Fransson et al. 2011). Acting as a dynamic reservoir, the micelles release PTX only after a concentration threshold is exceeded, resulting in disproportionate increases in area under the concentration–time curve (AUC) (Stage et al. 2018). The commonly administered dose of Taxol (CrEL-based PTX) is 175 mg/m^2^ delivered as 3-h IV infusion every 3 weeks (175 mg/m^2^, 3 h, q3w). This regimen leads to a maximum plasma concentration (C_max_) of 5.1 μM, a clearance of 12.0 L/h/m^2^, and a T_c_ > 0.05 μM of 23.8 h (Stage et al. 2018). CrEL-based PTX exhibits nonlinear pharmacokinetics with increasing dose. A 3-h infusion of 240 mg/m^2^ results in a higher C_max_ of 9.6 μM and decreased clearance of 4.8 L/h/m^2^ (Stage et al. 2018). Dose-dependent variability in clearance and volume of distribution contributes to unpredictable interpatient pharmacokinetic responses and complicates dose optimization (El Nemr et al. 2018).

### Recommended therapeutic exposure range for paclitaxel from Taxol

Pharmacokinetic parameters, including C_max_ and AUC, are associated with clinical outcomes; however, T_c_ > 0.05 μM remains the most consistently validated predictor of toxicity and overall clinical outcomes (Hertz et al. 2024). Some studies report correlations between clinical outcomes and C_max_ measured at the end of infusion, a time point more practical for routine clinical implementation. However, the association between C_max_ and clinical outcomes remains insufficiently validated and requires confirmation in large, well-conducted studies before translation into clinical practice can be considered (van Hagen et al. 2012; Crombag et al. 2019). AUC is associated with clinical activity, including survival; however, its utility remains limited (Henningsson et al. 2001; Hertz et al. 2024). T_c_ > 0.05 μM or T_c_ > 0.1 μM is associated with therapeutic response. For example, patients with T_c_ > 0.1 μM higher than 15 h show a 1-year survival of 28%, while patients with T_c_ > 0.1 μM shorter than 15 h show a 1-year survival of 12% (Huizing et al. 1997). Furthermore, strong evidence shows that higher PTX exposure, as estimated using T_c_ > 0.05 μM or T_c_ > 0.1 μM, is associated with a greater risk of neutropenia and neuropathy (Hertz et al. 2024). Based on these efficacy and toxicity findings, Joerger et al. recommend PTX T_c_ > 0.05 μM values of 26–30 h for conventional 3-h PTX infusions combined with a platinum agent, for therapeutic dose adjustment and for toxicity monitoring. Tc > 0.05 μM values exceeding 39 h are strongly associated with severe (grade 3/4) neutropenia (Joerger et al. 2017; Hertz et al. 2024). Given that conventional Taxol uses the same efficacy and safety index (T_c_ > 0.05 μM), PTX shows a narrow therapeutic window.

To improve clinical outcomes while reducing toxicity, PTX formulations should achieve exposure separation—maximizing drug uptake in tumor tissue while maintaining plasma T_c_ > 0.05 μM values within 26–30 h.

## Formulation strategy I: development of cremophor EL-free injectable formulations

### Albumin-bound nanoparticle formulation of paclitaxel

The first and most commercially successful CrEL-free IV formulation of PTX is the albumin-bound nanoparticle formulation, Abraxane. The FDA approved Abraxane in 2005 for metastatic breast cancer, followed by approvals for NSCLC and pancreatic cancer. Consequently, Abraxane is now used to treat solid tumors in approximately 70 countries (Table 2). Dosing ranges from 135 to 375 mg/m^2^, depending on cancer type (Kundranda and Niu 2015; Sarkar et al. 2023). In this formulation, six or seven PTX molecules bind noncovalently to albumin, forming primary PTX-albumin aggregates (4–14 nm), which subsequently associate into larger aggregates averaging 130 nm. These PTX-albumin nanoparticles rapidly dissociate in the bloodstream (Sofias et al. 2017) (Fig. 2).

Specifically, beyond eliminating CrEL-related toxicity, Abraxane introduces a mechanism for enhanced tumor targeting (Ji et al. 2017). It leverages natural albumin transport pathways by binding gp60 receptors on endothelial cells, triggering caveolae-mediated transcytosis, and transporting the nanoparticles across the vascular wall into the tumor interstitium (Giordano et al. 2017). Furthermore, secreted protein acidic and rich in cysteine (SPARC), often overexpressed in the tumor microenvironment, may bind albumin and retain PTX within tumors, prolonging its local exposure. Consequently, this interaction may enhance gp60/caveolae-mediated transcytosis and increase tumor drug accumulation. However, clinical evidence supporting SPARC as a predictive biomarker of Abraxane efficacy remains inconsistent (Hidalgo et al. 2015). While gp60-mediated transport and SPARC-associated retention provide a plausible biological rationale, the inconsistency of SPARC as a predictive biomarker limits its use for patient selection (Hidalgo et al. 2015; Zhou et al. 2021). Overall, the clinical value of Abraxane primarily arises from excipient elimination and improved administration feasibility, rather than consistently reproducible tumor-targeting mechanisms (Li et al. 2021a, b).

A multicenter Phase II trial of 43 patients with advanced NSCLC treated with 260 mg/m^2^ as a 30-min infusion without premedication demonstrates the clinical activity of Abraxane. The formulation exhibits an ORR and disease control rate of 16% and 49%, respectively. No severe hypersensitivity reactions are observed, and 95% of patients tolerated the full dose without reduction, confirming that Abraxane effectively overcomes the limitations associated with Taxol (Green et al. 2006). Large Phase III trials confirm that Abraxane maintains a manageable safety profile even when combined with cytotoxic agents such as carboplatin or gemcitabine, with reduced severe neuropathy and hypersensitivity compared with CrEL-based formulations (Table 3). In metastatic breast cancer, Gradishar et al. report that Abraxane (260 mg/m^2^ q3w) results in a significantly lower rate of grade 4 neutropenia than CrEL-based PTX (9% vs. 22%). Severe hypersensitivity reactions are absent (0% vs. 2%), but grade 3 sensory neuropathy occurs more frequently in Abraxane (10% vs. 2%) (Gradishar et al. 2005). The MTD of Abraxane is 300 mg/m^2^ over a 0.5-h IV infusion, with neutropenia as the DLT (Gardner et al. 2008). Toxicity profile shift of Abraxane toward increased peripheral neuropathy but reduced neutropenia seems to reflect tissue distribution characteristics of Abraxane.

**Table 3:** Clinical PTX formulations developed to overcome CrEL-related hypersensitivity

Formulation (Route)	Patient	dose/schedule	Clinical outcome/ Grade 3/4 toxicity	DLT profile	TI Interpretation
Abraxane (IV)	Metastatic breast cancer	Deliverable dose: 260 mg/m^2^ q3w (30 min infusion) MTD: 300 mg/m^2^ (30-min infusion)	ORR ↑ (33% vs Taxol 19%) Neutropenia (9%); sensory neuropathy (10%)	HSR ↓↓↓ / Neuropathy ↔ (↑) / Neutropenia ↔ (↓)	CrEL-related HSR solved, but neutropenia remained as intrinsic paclitaxel DLT TI 300/260 = 1.15
Lipusu (IV)	Advanced NSCLC	Deliverable dose: commonly 175 mg/m^2^ q3w (or weekly split) in combination regimens MTD: not reported	ORR 26%, PFS 5.1 months, OS 9.0 months (vs Taxol ORR 24%, PFS 4.2 months, OS 9.3 months) peripheral neuropathy 8% (vs Taxol 28%)	HSR/infusion burden↓↓ / Neuropathy ↓/ Neutropenia ↔	CrEL-related HSR solved
Genexol-PM (IV)	Metastatic breast cancer	Deliverable dose: 260 mg/m^2^ MTD: 390 mg/m^2^ q3w	ORR ↑ (38.0% vs Taxol 24.3%) Sensory neuropathy (↑); myalgia (↑) (vs Taxol)	HSR ↓↓↓ / Neuropathy ↔ (↑) / Neutropenia ↔ (↓)	CrEL-related HSR solved, but neutropenia remained as intrinsic paclitaxel DLT TI 390/260 = 1.5
Nanoxel (IV)	Advanced/metastatic breast cancer	Nanoxel 300 mg/m^2^ q3w × 6 cycles vs Taxol 175 mg/m^2^ q3w MTD 375 mg/m^2^	ORR: 40% vs (vs Taxol 31%) Neutropenia 56.3% (vs Taxol 50%) Neuropathy 12.5% (vs Taxol 6.3%)	HSR/infusion reactions ↓↓↓ /Neutropenia ↔ /Neuropathy ↔ (high-dose ↑)	CrEL-related HSR solved, but neutropenia remained as intrinsic paclitaxel DLT TI 375/300 = 1.25
Apealea (IV)	Relapsed platinum-sensitive ovarian cancer	Deliverable dose: 250 mg/m^2^ q3w MTD: 250 mg/m^2^	Non-inferior PFS vs Taxol Neutropenia (Grade 3/4); neuropathy (Grade 3/4)	HSR ↓↓↓ / Neuropathy ↔ / Neutropenia ↔	CrEL-related HSR solved, yet efficacy/therapeutic index expansion was marginal

*DLT* dose-limiting toxicity, *HSR* hypersensitivity reaction, *IV* intravenous, *TI* therapeutic index, *NSCLC* non-small cell lung cancer, *ORR* objective response rate, *OS* overall survival, *PFS*, progression-free survival, *MTD* maximum tolerated dose, *q3w* every 3 weeks

Chen et al. (2014) reported that a Tc > 0.05 μM is not significantly correlated with neutropenia induction in Abraxane treated patients. Rather, the probability of a ≥ 50% reduction in absolute neutrophil count is best correlated with the duration above a PTX concentration of 0.84 μM (i.e., Tc > 0.84 μM). Less-frequent neutropenia with Abraxane may result from the rapid decline of PTX concentrations below the plasma threshold of 0.84 μM (Chen et al. 2014; Li et al. 2021a, b).

Following IV infusion of Abraxane, PTX exhibits linear kinetics across the 135–300 mg/m^2^ dose range, independent of the infusion duration (0.5 h vs 3 h). In a comparative pharmacokinetic study of Abraxane (260 mg/m^2^ over 0.5-h IV infusion) and Taxol (175 ng/m^2^ over 3-h IV infusion), Abraxane shows a 53% larger volume of distribution (632 L/m^2^) and 43% higher total clearance (15 L/h/m^2^), with a similar elimination half-life of ~ 21 h (Sarkar et al. 2023). These findings suggest that Abraxane demonstrates more rapid and deeper tissue penetration compared with Taxol by increasing unbound PTX concentration while CrEL reduced PTX distribution by entrapping PTX into CrEL micelle. These observations align with clinical data showing that the unbound PTX concentration inversely correlates with circulating CrEL concentration (Henningsson et al. 2003).

Collectively, the development of the albumin-bound nanoparticle formulation results in altered pharmacokinetics and tissue distribution properties. This formulation separates the DLT (Tc > 0.84 μM) from the clinical outcome index (Tc > 0.05 μM), thereby expanding the therapeutic index of PTX compared with Taxol.

### Liposomal formulation of paclitaxel

Lipusu is the first liposomal PTX formulation approved in China for the treatment of ovarian cancer, breast cancer, and NSCLC (Table 2). PTX was encapsulated by liposomes of 400 nm in diameter, composed of lecithin and cholesterol (Wang et al. 2017). In advanced NSCLC, Lipusu achieves an ORR of 26%, with median OS of 9.0 months (vs. ORR 24% and OS 9.3 months for the Taxol group). A notable tolerability signal is the lower incidence of peripheral neuritis (8% vs. 28% in the Taxol group) (Xu et al. 2013). Lipusu shows linear pharmacokinetics with increased stability. Lipusu relies primarily on the enhanced permeability and retention (EPR) effect, where liposomes accumulate in leaky tumor vasculature. The probability of neutropenia is correlated with PTX exposure (Zhou et al. 2021).

Collectively, these data support interpreting Lipusu primarily as a CrEL-free injectable formulation with reduced infusion time, while acknowledging that PTX-intrinsic toxicities (myelosuppression/neuropathy under combination dosing) continue to define the practical therapeutic ceiling unless exposure separation is clearly demonstrated.

### Polymeric micelle formulation of paclitaxel

Genexol-PM is polymeric micelle formulation, approved in the Republic of Korea in 2006, to treat metastatic/recurrent breast cancer and NSCLC (Table 2). It is based on biodegradable methoxy poly(ethylene glycol)–poly(lactide) (mPEG-PLA) block copolymers that self-assemble into 25 nm micelles, encapsulating PTX in a hydrophobic core. The hydrophilic PEG shell stabilizes the nanoparticles in aqueous solution, allowing IV administration without CrEL and reducing associated toxicities (Kim et al. 2004; Werner et al. 2013) (Fig. 2).

In a Phase I study of patients with advanced solid tumors, Genexol-PM was administered as a 3-h IV infusion without steroid premedication. The MTD was 390 mg/m^2^, substantially higher than the 175 mg/m^2^ standard for CrEL-based PTX. The therapeutic dose was 260 mg/m^2^, indicating an improved therapeutic index. DLTs included peripheral neuropathy, myalgia, and neutropenia, with no acute hypersensitivity reactions typically associated with CrEL. Pharmacokinetic analysis shows approximately linear, dose-proportional exposure, confirming that Genexol-PM overcomes the pseudo-nonlinear pharmacokinetics of CrEL-based PTX (Kim et al. 2004). In a Phase II study, 41 patients with metastatic breast cancer were administered Genexol-PM at 300 mg/m^2^ q3w. The formulation demonstrates notable anti-tumor activity, with an ORR of 58.5%. Hypersensitivity reactions are observed in 19.5% of patients (Grade 3 in 4.9%) despite the solvent-free vehicle; however, these events are manageable and mechanistically distinct from CrEL-induced reactions (Lee et al. 2007). The Genexol-PM arm (260 mg/m^2^) demonstrates a significantly higher ORR (38.0% vs. 24.3%, p = 0.002) while progression free survival (PFS) and OS are not significantly different in in patients with metastatic breast cancer compared with CrEL-based PTX (175 mg/m^2^) (Park et al. 2017) (Table 3). While the improved ORR supports clinical activity, the absence of a statistically significant PFS/OS benefit suggests that dose escalation enabled by a CrEL-free carrier may be insufficient to alter long-term disease trajectory in heterogeneous tumors. This discrepancy may reflect limitations of response-based endpoints when exposure is uneven or insufficiently sustained across lesions. Therefore, CrEL-free should be considered a necessary but insufficient criterion; clinically meaningful therapeutic index expansion is more likely when supported by demonstrable exposure separation and/or sustained intratumoral availability of pharmacologically active PTX.

Nanoxel is a CrEL-free polymeric micellar formulation approved in India and initially used for metastatic breast cancer (Table 2). In early dose-escalation studies, Nanoxel was administered as a 1-h IV infusion q3w without premedication across 135–375 mg/m^2^, showing dose-exposure proportionality. An MTD of Nanoxel is reported to be 375 mg/m^2^ (Barkat et al. 2019). In a subsequent Phase II evaluation following anthracycline failure, Nanoxel was tested at 300 or 220 mg/m^2^ q3w versus Taxol 175 mg/m^2^ q3w. Efficacy is comparable, with ORR 38–40% at both Nanoxel doses compared with 31% for Taxol. Hematologic toxicity remains dose-dependent: neutropenia occurs in 39.4% (220 mg/m^2^) and 56.3% (300 mg/m^2^) versus 50% for Taxol, while grade 3 sensory neuropathy occurs in 12.5% (300 mg/m^2^) versus 6.3% for Taxol. Consistent with the CrEL-free design, real-world further reports no hypersensitivity reactions across 596 infusions (Ranade et al. 2013). Within the therapeutic index framework, Nanoxel therefore represents a deliverability/vehicle-toxicity relief strategy that enables higher feasible dosing and reduces infusion reactions, while intrinsic PTX DLTs remain exposure- and dose-dependent constraints.

### Micellar formulation of paclitaxel

The EMA authorized Apealea, a water-soluble micellar formulation of PTX, on November 20, 2018, for the treatment of adult patients with first relapse of platinum-sensitive ovarian, peritoneal, or fallopian tube cancer in combination with carboplatin (Table 2). The formulation comprises biodegradable 20–30 nm micelles formed with two vitamin A derivatives, which rapidly disassemble upon dilution, enabling CrEL-free delivery and eliminating Cremophor-related hypersensitivity (Borgå et al. 2019; Vergote et al. 2020) (Fig. 2).

In a first-in-human Phase I dose-escalation trial, 34 patients with recurrent advanced solid tumors—including ovarian, melanoma, colon, and uterine cancers—were administered Apealea at 90–275 mg/m^2^ (1 h infusion, q3w, up to 3 cycles). The MTD was established at 250 mg/m^2^. Grade ≥ 3 toxicities included neutropenia (grade 3: 51.2%; grade 4: 17.1%), leukopenia (grade 3: 39.0%), and sensory peripheral neuropathy (grade 3: 51.2%) (Borgå et al. 2019). Apealea (250 mg/m^2^ over 1-h IV infusion) achieves a PFS and OS of 10.3 and 25.7 months, respectively, indicating non-inferiority in efficacy while offering reduced CrEL-related hypersensitivity and shorter infusion time compared with Taxol (175 mg/m^2^ over 3-h IV infusion), with carboplatin (AUC 5–6 min∙mg/mL) administered 30 min later, q3w for up to 6 cycles in 789 patients with first relapse of platinum-sensitive ovarian, peritoneal, or fallopian tube cancer. However, higher rates of serious hematological toxicities are observed with Apealea (41%) than with Taxol (27%) (Vergote et al. 2020) (Table 3). To elucidate the peripheral neuropathy development, the comparative pharmacokinetics and tissue distribution of three PTX formulations—Taxol, Abraxane, and Apealea—were investigated in rats. Taxol exhibits approximately two-fold lower unbound PTX concentrations but similar total concentrations compared with Abraxane and Apealea, which is similar to the clinical case (Girdenytė et al. 2024). Both Apealea and Abraxane demonstrate significantly higher distribution to skeletal muscle, sciatic nerve, and dorsal root ganglia than Taxol. However, no differences in tissue distribution are observed between the Abraxane and Apealea arms across the tissues (Girdenytė et al. 2024). These findings indicate that CrEL-free formulations eliminate CrEL-induced hypersensitivity but can lead to higher PTX exposure at peripheral neuropathy sites in comparison to Taxol. Despite receiving regulatory approval in 2023, Apealea is no longer authorized for sale in the EU (Table 2).

## Formulation strategy II: next-generation paclitaxel formulations under clinical investigation

Approved CrEL-free injectable formulations, including Abraxane, Apealea, and Genexol-PM, successfully eliminate the need for steroid premedication and reduce hypersensitivity reactions; however, severe myelosuppression remains an intrinsic DLT of PTX (Vishnu et al. 2011). This underscores a key principle: the therapeutic potential of PTX is constrained by its toxicity profile. DLTs do not merely represent adverse effects; they define the maximal tolerable threshold of clinical efficacy and often necessitate dose reductions, treatment delays, or discontinuation, potentially compromising long-term disease control and patient survival (Lyman 2009). Accordingly, novel PTX nano-formulations need to be designed to modify biodistribution—enhancing tumor tissue accumulation while limiting drug exposure in sensitive tissues such as peripheral nerves and bone marrow—have undergone clinical evaluation (Table 4).

**Table 4:** PTX formulations under clinical evaluation for DLT mitigation

Formulation (Route)	Technology	Primary goal / mechanism	DLT profile	Clinical outcome
NK105 (IV)	Polymeric micelle	Increased plasma exposure but decreased distribution to peripheral nerves or dorsal root ganglia	DLT was Neutropenia MTD 150 mg/m^2^ Dose 65 mg/m^2^, q1w TI 2.31	Comparable efficacy to Taxol Grade ≥ 3 neuropathy 0% (vs Taxol 9.8%) More frequent neutropenia
Paclitaxel Poliglumex (IV)	Macromolecular conjugate (poly-L-glutamic acid)	Prolong systemic half-life and enhance tumor-selective drug accumulation	MTD not identified Dose 175 mg/m^2^, q3w combination with carboplatin	Comparable OS and DCR compared with Taxol Reduced neutropenia and neuropathy Need to further clinical evaluation
LEP-ETU (IV)	Liposome	Enhance tolerability to permit higher dose intensity EPR effect	DLTs were febrile neutropenia and neuropathy MTD 375 mg/m^2^ Dose 275 mg/m^2^ TI 1.34	↑Enabled higher doses, but intrinsic DLTs persisted
EndoTAG-1 (IV)	Cationic liposome	Target the negatively charged tumor endothelium	Formulation-specific hypersensitivity reactions	Failure (Efficacy) in triple negative breast cancer patients (Phase III trial) Formulation-specific hypersensitivity reactions

*PTX* paclitaxel, *DLT* dose-limiting toxicity, *MTD* maximum tolerated dose, *IV* intravenous

### Polymeric micelle–based paclitaxel formulation NK105

NK105, a polymeric micelle–based formulation, is developed to mitigate PTX-induced peripheral neuropathy. Novel amphiphilic block copolymers are engineered with polyethylene glycol (PEG) as the hydrophilic component and modified polyaspartate as the hydrophobic core, which self-assemble to encapsulate PTX within the micelle inner core in an aqueous medium. NK105 micelles have a median diameter of 85 nm. Preclinical studies report higher PTX concentrations in the plasma and tumor tissue following IV administration of NK105, with reduced clearance compared with free PTX. The tumor AUC of NK105 is 25-fold higher than that of free PTX. Repeated administration of NK105 to rats at 7-day intervals results in fewer toxic effects on peripheral nerves compared with free PTX. These findings suggest enhanced antitumor activity with reduced neurotoxicity, potentially owing to diminished drug distribution to the dorsal root ganglion (Hamaguchi et al. 2005; Nakamura et al. 2017).

In a randomized Phase II study of patients with advanced/recurrent breast cancer, NK105 demonstrates comparable efficacy to Taxol while reducing neurotoxicity. Grade ≥ 3 peripheral sensory neuropathy occurs in 0% of NK105 recipients versus 9.8% in the Taxol arm, and time to neuropathy onset is prolonged (2.6 vs 1.2 months; p = 0.001). Neutropenia occurs more frequently with NK105 than with Taxol, but most patients do not require granulocyte-colony stimulating factor or dose reductions (Kosaka et al. 2022). In the Phase III PEARL trial, NK105 (65 mg/m^2^, q1w, up to 3 cycles) maintains a favorable peripheral neuropathy profile but does not demonstrate superiority in PFS compared with conventional PTX (80 mg/m^2^, q1w, up to 3 cycles) (Fujiwara et al. 2019). Based on these results, neutropenia is the DLT of NK105 (Kato et al. 2013) (Table 4).

Given that plasma PTX exposure from NK105 is 15-fold higher than conventional PTX, the incidence of neutropenia may increase. However, the limited distribution of NK105 to peripheral nerves or dorsal root ganglia, as reported in rats (Hamaguchi et al. 2005; Nakamura et al. 2017), may confer an advantage over Taxol in reducing peripheral neuropathy. However, this safety trade-off should be interpreted cautiously, as reduced peripheral neuropathy through dose reduction may coincide with insufficient pharmacologically active PTX in tumors unless the PTX formulation showed tumor-specific accumulation. Thus, successful translation requires preserving adequate intratumoral availability of free PTX at clinically feasible dose intensity through the active tumor targeting technique.

### Paclitaxel-polymer conjugate poliglumex

PTX-Poliglumex, a conjugate of PTX and poly-L-glutamic acid with a 1:11 stoichiometry (molecular weight of 48 KDa), forms a water-soluble formulation. In this system, inactive PTX enters the tissue via endocytosis, and active PTX is subsequently released by the lysosomal enzyme cathepsin B. Approximately 1–2% of the total AUC of PTX Poliglumex is pharmacologically active free PTX and exhibits a prolonged half-life. Moreover, preclinical studies demonstrate that PTX Poliglumex accumulates 11-fold more in tumor tissue, where it gradually releases the active PTX (Singer 2005). Subsequent clinical studies using PTX Poliglumex at 175 mg/m^2^ as a 10–20 min infusion q3w reveal a favorable hematological toxicity profile in chemotherapy-naïve patients with advanced NSCLC. Grade 3/4 neutropenia and anemia are reduced in the PTX Poliglumex treatment group, and grade 3 neuropathy occurs in only 3% of patients (O’Brien et al. 2008). However, in a pivotal Phase III trial involving 400 patients with NSCLC, PTX Poliglumex in combination with carboplatin did not demonstrate superiority in OS compared with the standard PTX–carboplatin regimen. One-year survival rates and DCR are also comparable between the treatment arms. Grade 3/4 neuropathy is not significantly different between the PTX Poliglumex/carboplatin group and conventional PTX/carboplatin groups. Time to first manifestation of neuropathy is delayed in the PTX Poliglumex arm. Additionally, safety analysis reveals a similar outcome and lower rates of neuropathy and neutropenia for the 175 mg/m^2^ compared with the 210 mg/m^2^ dose. Therefore, 175 mg/m^2^ of PTX Poliglumex is recommended for further clinical studies (Langer et al. 2008) (Table 4).

### Liposome-entrapped paclitaxel

Liposome-entrapped PTX Easy-to-use (LEP-ETU) is a lyophilized liposomal formulation composed of negatively charged synthetic phospholipids and cholesterol. Upon reconstitution, it forms unilamellar vesicles with a mean particle size of 150 nm and an entrapment efficiency > 90% (Zhang et al. 2005). LEP-ETU was designed to utilize the EPR effect in tumor tissues due to leaky vasculature and poor lymphatic drainage.

A multi-institutional Phase I study escalates the LEP-ETU dose to levels exceeding the standard Taxol dose of 175 mg/m^2^. The MTD for LEP-ETU administered q3w was established at 325 mg/m^2^ (Fetterly et al. 2008). In a randomized two-period crossover bioequivalence study of 58 patients with advanced cancer, the C_max_ and AUC of PTX from LEP-ETU (175 mg/m^2^, 3 h IV infusion) are equivalent to those of Taxol (Slingerland et al. 2013). Pharmacokinetics/pharmacodynamic modeling and simulation results reveal that LEP-ETU 325 mg/m^2^ q3w provides an acceptable rate of neutropenic events relative to that observed with conventional Taxol (175 mg/m^2^). A 275-mg/m^2^ dose may offer an improved therapeutic index (Fetterly et al. 2008). LEP-ETU formulation demonstrates anti-tumor activity, yielding partial response rate and stable DCR of 12% and 44%, respectively, in a heavily pretreated patient population (Damjanov et al. 2005) (Table 4). The current LEP-ETU formulation shows similar pharmacokinetic properties and is expected to produce a comparable EPR effect relative to Taxol. Therefore, clinical advantage of LEP-ETU over Taxol in terms of clinical efficacy and intrinsic toxicity seems to be limited.

### Cationic liposomal paclitaxel endoTAG-1

EndoTAG-1 represents a more active targeting strategy designed to alter the PTX biodistribution. EndoTAG-1 consists of PTX embedded in positively charged liposomes, engineered to electrostatically bind specific cells. This interaction concentrates PTX at sites of tumor angiogenesis, where newly formed tumor blood vessels display abundant negatively charged endothelial cells on their surface (Eichhorn et al. 2010). This strategy functions as a tumor vascular-targeting approach, disrupting tumor vasculature to inhibit tumor growth and metastasis, thereby enhancing efficacy while minimizing systemic exposure (Strieth et al. 2008; Eichhorn et al. 2010). A randomized Phase II study in advanced triple-negative breast cancer compares the efficacy of EndoTAG-1 plus PTX with that of PTX monotherapy. In the per-protocol population, the combination therapy arm shows a higher clinical benefit rate (53% vs. 36%) and a positive trend toward increased OS (15.1 vs. 8.9 months) (Awada et al. 2014). However, an independent feasibility study in patients with HER2-negative breast cancer reports that some patients discontinue treatment owing to grade 3 hypersensitivity reactions specifically to the EndoTAG-1 formulation, despite premedication. This finding indicates that, while advanced formulations mitigate certain toxicities, they may introduce novel safety considerations requiring careful management (Ignatiadis et al. 2016). The clinical development of EndoTAG-1 encountered a significant setback following subsequent results obtained from its pivotal Phase III MAJESTIC trial. The study evaluates EndoTAG-1 combined with PTX versus PTX alone in patients with advanced triple-negative breast cancer. The trial fails to achieve its primary endpoint, with no statistically significant improvement in PFS observed in the EndoTAG-1 combination arm (Ignatiadis et al. 2016) (Table 4). This outcome highlights the translational gap between “active targeting” observed in preclinical models and its performance in clinical settings. While electrostatic targeting of tumor endothelial cells via cationic surface charge is mechanistically sound in vitro, the formation of a protein corona in the protein-rich human bloodstream can shield or neutralize the cationic surface, thereby diminishing targeting efficiency (Li et al. 2021a, b). Furthermore, reliance on vascular targeting alone may be insufficient to eradicate poorly vascularized tumor cores. Thus, the limited clinical success of these formulations suggests that future physicochemical targeting strategies must consider the complex biological identity (e.g., protein corona) that nanoparticles acquire in systemic circulation, rather than relying solely on their synthetic design.

### Lessons from paclitaxel formulations under clinical development

The overall clinical development of next-generation PTX formulations—including polymeric micelles (e.g., NK105) and liposomal platforms (e.g., LEP-ETU and EndoTAG-1)—highlights a key translational challenge: the unpredictability of efficiency of tumor targeting in humans (Awada et al. 2014; Seyam et al. 2025). While these formulations are designed to exploit prolonged circulation for preferential tumor accumulation via the EPR effect or electrostatic targeting, clinical evidence suggests that the EPR effect in human solid tumors is considerably more heterogeneous than in murine models (Bae and Park 2020; Izci et al. 2021). Physiological barriers, such as dense stroma tissue and elevated interstitial fluid pressure, frequently hinder nanoparticle penetration (Fu et al. 2021). Therefore, extended systemic circulation does not guarantee sufficient intratumoral exposure (Izci et al. 2021). Consequently, while these platforms successfully improve tolerability by reducing vehicle-related toxicities—thereby increasing the maximum tolerated concentration—they often fail to consistently enhance tumor-site efficacy, limiting the achievable effective dose. Taken together, relying solely on passive EPR-mediated accumulation is insufficient to achieve a meaningful therapeutic index expansion. Instead, future formulation strategies should be assessed based on their ability to mechanistically overcome tumor-access and intratumoral transport limitations (Lu and Prakash 2021). This focuses formulation design from merely extending systemic circulation to strategies that actively exploit tumor microenvironment modulation or transcytosis-mediated entry pathways.

## Formulation strategy III: advanced targeted formulations under preclinical stage

Recent preclinical active platforms aim to expand the therapeutic index via adding mechanism-triggered delivery, rather than depending solely on prolonged circulation or EPR effect. The common biopharmaceutical goal is to keep PTX largely inactive systemically—thereby minimizing peak-related toxicity—while promoting selective activation or uptake within tumor-relevant compartments (Table 5).

**Table 5:** Representative recent preclinical PTX formulation for active tumor targeting

Name/Route	Technology	Primary goal/mechanism	Toxicity	Results	Translational notes
Albumin-hitchhiking paclitaxel prodrug nanoparticles (PC NPs → PC@albumin) /IV	Noncovalent binding to serum albumin, enabling ultrasmall PC@albumin complex formation redox-cleavable disulfide linker	Spontaneous albumin hitchhiking in situ size conversion Redox-cleavable prodrug activation	Excipient-free, albumin-assisted transport; off-target exposure↓ Redox-gated activation; systemic active paclitaxel ↓	Size/ζ: PC NPs 77.1 ± 0.6 nm; ζ − 15.9 ± 0.5 mV Serum conversion with albumin → PC@albumin 3.1 ± 0.5 nm In vivo efficacy: PTX 30 mg/kg → 93% of tumor inhibition	Translation will depend on robust albumin association/size conversion and the extent of interpatient redox variability
Cysteine-activated paclitaxel prodrug nanoparticles /IV	formulated with DSPE-PEG2000 (thin-film hydration) Cysteine-responsive design enabling trigger-dependent activation in the tumor microenvironment	Cysteine-triggered activation Fast release after activation intracellular paclitaxel ↑	Trigger-restricted activation; systemic active- paclitaxel ↓	Loading: 20.8 ± 0.9% Size/ζ: 142 ± 2.3 nm/ − 26.3 ± 3.7 mV Stability: stable for 1 week at 4 °C Release: Fast release after cysteine activation > 90% within 6 h	Clinical translation will hinge on trigger availability across tumor microenvironments and tightly controlled prodrug synthesis/activation kinetics under CMC
CD47p/AZE-Paclitaxome-2 /IV	sphingolipid-derived nanovesicle sphingomyeline was covalent bound to paclitaxel with ester, disulfide, or thioketal linkage	CD47 to evade phagocytic clearance and extend circulation time AZE for tumor penetration Redox-cleavable prodrug activation	Long circulation penetration of tumor microenvironment Redox-gated activation; systemic active paclitaxel ↓	Size/ζ: 100 nm/ − 30 mV Stability: stable for 2 wk at 4 °C Near-complete inhibition of primary tumor MTD↓: 70–100 mg/kg (vs taxol 20 mg/kg) No myelosuppression or DRG toxicity reported	Paclitaxome aims to outperform Abraxane by providing more sustained and targeted treatment
Folated coated hybrid exosomes /IV	MSC-derived exosomes fused with folate-modified cationic liposomes (Liposome:Exosome = 1:4, v/v) Hybrid exosome–liposome vesicles formed via membrane fusion	Paclitaxel loading/stability↑ Folate-mediated targeting Sustained release	Sustained release; peak-driven toxicity risk↓ Near-neutral ζ; nonspecific interactions↓	Loading: 2.2% Size: ~ 150 nm/ − 1.4 mV Stability: no size change at 4 °C, 26 weeks Release: sustained for 48 h	Key barriers include exosome source heterogeneity and scalable, standardized manufacturing
Polymer-coated magnetic paclitaxel nanoparticles/IV	SPION core sequentially coated with chitosan, paclitaxel, PEG, and folate Layer-by-layer surface engineering to yield folate-decorated magnetic paclitaxel nanoparticles	Folate-targeted SPION platform; tumor uptake↑ Magnetic core; guidance potential	Targeted uptake; dose-sparing Reduced nonspecific distribution; off-target toxicity↓	Size: 31.89 ± 6.9 → 63.3 ± 12.55 → 102.6 ± 17.66 nm ζ: − 21.8 ± 5.8 → − 14.0 ± 6.16 → − 47.5 ± 7.36 mV Biocompatibility: high concentrations of SPIONs can induce oxidative stress or mitochondrial dysfunction. SPION carrier > 90% viability at 30 μg (highest amount tested)	Translational feasibility is constrained by long-term SPION clearance/safety and the practicality of magnetic guidance Consistent performance will require scalable, reproducible coating and consideration of folate-receptor heterogeneity

*PTX* paclitaxel, *IV* intravenous, ζ, zeta potential, *DSPE–PEG2000* Distearoylphosphatidylethanolamine-polyethylene glycol 2000, *CMC* critical micelle concentration, *CD47p*, *CD47*-derived self peptide 4, *AZE* azepane, *MTD* maximum tolerated dose, *DRG* dorsal root ganglion, *MSC* mesenchymal stem cell, *SPION* superparamagnetic iron oxide nanoparticle

### Albumin-hitchhiking paclitaxel prodrug nanoparticles

PTX is linked to albumin-binding ligands—such as fatty acids (e.g., stearic acid)—via stimuli-responsive linkers (e.g., disulfide bonds) to form PTX prodrugs. These prodrugs spontaneously self-assemble into stable PTX prodrug nanoparticles (PC NPs) in aqueous solution, exhibiting a size of 77.1 ± 0.6 nm and a zeta potential of -15.9 ± 0.5 mV. Upon IV administration, these nanoparticles encounter abundant serum albumin and rapidly convert into prodrug–albumin complexes (PC@albumin) with a size of 3.1 ± 0.5 nm, through non-covalent interactions between fatty acid ligands and albumin (Table 5). These complexes leverage the prolonged circulatory half-life of albumin (~ 19 days) to persist in the bloodstream, thereby avoiding rapid renal or hepatic clearance. Circulating PC NPs can also exploit albumin-specific receptors that are overexpressed in many cancers. They remain stable in the blood but disassemble within tumor cells in response to high GSH levels, releasing active PTX directly into tumor tissue. In 4T1 models (30 mg/kg), this PC NP platform achieves 93% tumor inhibition, suggesting that albumin-mediated transport successfully limits off-target exposure (Lu et al. 2025).

Regarding scale-up manufacturing, the albumin-hitchhiking system employs chemically defined prodrugs, enabling high-yield and cost-effective production. This system self-assembles with serum albumin, providing the scalability and reproducibility necessary for clinical translation (Pei et al. 2023). However, the translational viability of this approach depends on the overexpression of gp60 or SPARC and the consistency of the redox-responsive mechanism across diverse physiological conditions. Premature cleavage of the prodrug could result in systemic PTX release, thereby negating the safety benefits offered by the albumin-shielded transport.

### Cysteine-activated paclitaxel prodrug nanoparticles

Distearoylphosphatidylethanolamine–polyethylene glycol 2000 (DSPE–PEG2000) functions as an amphiphilic stabilizer, creating a hydrophilic shell around the hydrophobic PTX prodrug core. This enhances stability, minimizes premature drug release, and decreases immune system clearance. Upon reaching the tumor microenvironment, which features higher cysteine concentration, the disulfide bond undergoes cleavage, releasing active PTX directly into tumor cells. This platform aims to suppress systemic exposure to active PTX by restricting drug release to cysteine-rich environments. This approach is designed to enhance the therapeutic index by ensuring that the cytotoxic payload is activated by the unique chemical signature of the tumor, rather than by physical accumulation (Haddad et al. 2022).

The DSPE–PEG2000 shell achieved efficient loading and rapid cysteine-responsive release (> 90% within 6 h; Table 5). A critical challenge remains whether cysteine concentrations and reaction kinetics are sufficiently consistent across tumor types and stages to ensure reliable activation. Furthermore, preventing unintended activation in off-target tissues with comparable thiol environments remains a primary safety concern, necessitating stringent control of prodrug synthesis and activation kinetics (Zhang et al. 2024).

### CD47p/azepane-coated sphingolipid-derived nanovesicle

Sphingomyelin was covalently conjugated with PTX via ester, disulfide, or thioketal linkages, then self-assembled with 3% cholesterol to form a nanovesicle (i.e., Paclitaxome) with an average size of 100 nm and zeta potential of -30 mV. This formulation remained stable for 2 weeks, maintaining size and zeta potential. An ultra-pH-sensitive probe, azepane (AZE), and a CD47-derived self-peptide 4 (CD47p) were incorporated onto the surface of the formulation. AZE enhances tumor accumulation and penetration via cationization-enabled, adsorption-facilitated transcytosis, while CD47p reduces phagocytic clearance and prolongs systemic circulation. It could respond to elevated levels of tumor-associated stimuli—hydrolases, glutathione, or reactive oxygen species—enabling controlled and timely release of PTX within the tumor microenvironment (Wang et al. 2025; Table 5).

CD47p/AZE-Paclitaxome-2 demonstrates significant improvements in safety and efficacy compared with Taxol (Wang et al. 2025). Preclinical studies in mice show that the MTD of CD47p/AZE-Paclitaxome-2 ranges from 70–100 mg/kg, representing a substantial increase over the MTD of Taxol (20 mg/kg). Even at high doses, CD47p/AZE-Paclitaxome-2 did not induce the severe systemic toxicities associated with Taxol, including myelosuppression and neurotoxicity. In an orthotopic model of triple-negative breast cancer, CD47p/AZE-Paclitaxome-2 prolongs median survival time to 72 days, surpassing the survival observed with Taxol (52 days) and Abraxane (51 days). This platform can be adopted to enable co-delivery of combination therapies through the covalent conjugation with PTX along with gemcitabine or carboplatin (Wang et al. 2023, 2025).

### Folated coated hybrid exosomes

Hybrid exosomes loaded with PTX are engineered by fusing mesenchymal stem cell–derived exosomes with folate-modified cationic liposomes to enhance PTX loading and optimize surface characteristics. This platform represents a shift in drug delivery control, enabling membrane-engineered regulation to achieve a sustained release profile. Mechanistically, the fusion strategy converts unstable, burst-prone vesicles into stable hybrid vesicles with an average size of ~ 150 nm (stable for 26 weeks at 4 °C) and a near-neutral surface charge (zeta potential -1.4 mV). These hybrid exosomes show a sustained release pattern over 48 h. Incorporation of folate enables targeting of cancer cells that overexpress folate receptors, thereby enhancing tumor-specific uptake (Table 5). In CT60-bearing mice, PTX-loaded hybrid exosomes achieve higher tumor targeting and significant tumor suppression (Wang et al. 2024). However, the translational bottleneck resides in the manufacturing complexity of exosome-based systems. Batch-to-batch variability in exosome source and the fusion process remains a critical barrier, as inconsistent membrane composition may compromise reproducibility of the intended release kinetics (Farah et al. 2026).

### Polymer-coated magnetic paclitaxel nanoparticles

Polymer-coated magnetic PTX nanoparticles employ a superparamagnetic iron oxide nanoparticle core sequentially coated with chitosan, PTX, PEG, and folate to generate ligand-decorated magnetic nanoparticles. This platform implements a folate-mediated active targeting strategy to enhance tumor cell uptake and retention (Gui et al. 2021). Mechanistically, the stepwise physicochemical assembly, which increases particle size from ~ 31.9 to ~ 102.6 nm, is intended to concentrate PTX at the target site, enabling dose sparing. This biopharmaceutical strategy aligns with the toxicological principle that minimizing nonspecific distribution reduces off-target exposure, as supported by reported carrier-only biocompatibility (> 90% viability at 48 h; Table 5).

While polymer-coated magnetic PTX nanoparticles demonstrate clear physicochemical evolution and targeting potential, their translational feasibility is limited by the complexity of the multicomponent system. Incorporating a magnetic core and multiple coating steps introduces long-term disposition and safety uncertainties that are independent of PTX. Ultimately, the success of this approach depends on whether the incremental uptake advantage from folate targeting outweighs the added manufacturing complexity and the inherent heterogeneity of folate receptor expression in clinical tumor settings (Farah et al. 2026).

Collectively, the platforms in Table 5 demonstrate that preclinical innovation has progressed from simple carrier modification to precise control of drug activation and tissue entry. A rigorous evaluation of these active systems requires measuring parameters that directly reflect their intended mechanisms—such as the ratio of carrier-bound to free PTX, early-phase systemic spikes, and trigger-dependent release kinetics. These metrics provide the necessary link between biopharmaceutical design and verifiable toxicological advantage, contributing to the expansion of the PTX therapeutic index.

## Formulation strategy IV: expansion to subcutaneous delivery formulation

### Subcutaneous administration of Taxol

Developing subcutaneous (SC) formulations for anticancer drugs has emerged as a major trend, driven by clear clinical advantages, including shortened administration times, lower initial plasma concentrations through the sustained releasing depot system, and reduced healthcare resource utilization.

Along with this trend, a preclinical study evaluated hypersensitivity reactions and toxicity after SC administration of CrEL-PTX in 21 dogs with advanced cancer (Table 6). Dogs received one to five SC doses of Taxol at 85–170 mg/m^2^ every 14 or 21 days. None of the dogs exhibits systemic or acute local hypersensitivity reactions. However, two dogs developed severe skin lesions at the injection site after the fourth dose at the same location. The sustained absorption results in severe grade 4 neutropenia in 50% of the dogs at a dose of 115 mg/m^2^, probably attributed to prolonged exposure of bone marrow cells. Among dogs with measurable disease, 64% achieve a partial response after the first treatment (Silva et al. 2015). The findings suggest that PTX administered via SC injection achieves comparable efficacy, does not induce hypersensitivity reactions, and causes minimal local injection-site toxicity, provided that repeated injections at the same location are avoided.

**Table 6:** Representative recent preclinical studies evaluating the feasibility of subcutaneous PTX delivery

Formulation (Route)	Model	Dose/schedule	Toxicity	Clinical outcome	Durability of SC injection
Taxol (SC)	Tumor-bearing dogs	115 mg/m^2^	Grade 4 neutropenia in 50% of dogs	Toxicity study: significant systemic toxicity via SC route	Conventional Taxol is not SC-feasible; vehicle/tissue exposure constraints dominate
Ptx-PAAm(SC)	MCF7 tumor–bearing mice	15 mg/kg	No local necrosis or injection-site toxicity threefold increased MTD (vs IV Taxol)	Bioavailability 84%; superior antitumor activity vs IV Taxol	SC delivery of Ptx–PAAm prodrug can be viable by inert PTX at injection site It can reduce systemic peak exposure while sustaining tumor-relevant exposure
mPEG–PCL hydrogel (SC)	B16F10 tumor–bearing mice	0.5, 1, 2 mg/mL	None reported	Sol-to-gel transition at body temperature; sustained release > 10 days; greater tumor growth inhibition vs IV Taxol	Depot-type SC strategies can reduce systemic peak exposure while sustaining tumor-relevant exposure
PN–NCs-Ts gel (SC; intratumoral injection)	H22 tumor–bearing mice	20 mg/kg	low side effect reported	68.8% tumor inhibition (TNBC xenograft); ^a^Ki-67 positive ratio reduced to 9.4% (vs 33.6% saline)	PN–NCs-Ts gel slowed PTX release / aligning with stronger tumor control (~ 68.8% inhibition)
PTX-NCS-gel (SC; post-surgery local smear)	4T1-luc tumor resection model	20 mg/kg	minimal systemic toxicity	PTX-gel relapsed after day 14 with > 90% recurrence Gelation 33.1 °C; 93.07% paclitaxel release within 6 h	PTX-NCS-gel achieved durable local control (no detectable recurrence vs > 90% relapse with PTX-gel) with suppressed lung metastasis

*PTX* paclitaxel, *SC* subcutaneous, *IV* intravenous, *TI* therapeutic index, *Ptx-PAAm* PTX–linker–polyacrylamide conjugate, *MTD* maximum tolerated dose, *mPEG–PCL* methoxy poly(ethylene glycol) -poly(ε-caprolactone) copolymer, *TNBC* triple-negative breast cancer, *PN–NCs-Ts gel* paclitaxel-loaded nanocapsules–thermosensitive gel, *PTX-NCS-gel* Paclitaxel nanocrystals gel

^a^Ki-67 positive ratio means the percentage of actively dividing cancer cells, indicating rapid growth and a potentially worse prognosis

In mice, Taxol administered IV reaches an MTD of 60 mg/kg, whereas SC administration achieves 90 mg/kg, probably due to a reduced C_max_ compared to IV delivery (Bordat et al. 2022). OncoGel—a biodegradable hydrogel formulation containing PTX—exhibits sustained drug release and a comparable number of long-term survivors as IV Taxol after a single SC injection, despite being administered at one-tenth of the IV Taxol dose (Zhao et al. 2021). Collectively, SC administration of PTX using a depot system may represent a meaningful progress by sustaining tumor-relevant exposure above the MEC while reducing C_max_ through formulation and route-of-administration optimization.

### Paclitaxel–polyacrylamide prodrug conjugate

PTX–linker–polyacrylamide (Ptx–PAAm) is a polymer prodrug with PTX covalently conjugated to PAAm. PAAm is an uncharged, highly water-soluble, and biocompatible polymer with stealth characteristics. In this construct, PTX is attached to the polymer via a cleavable moiety at the C2’ hydroxyl group, thereby maintaining PTX in a pharmacologically inert state at the SC injection site and mitigating prolonged local exposure that can result in severe tissue necrosis. Ptx–PAAm adopt hydrazone linker that is stable at neutral pH but cleaved at pH 4.5–5.5 or disulfide linker. They remain stable in the blood but disassemble within tumor cells in response to low pH or high GSH levels, releasing active PTX directly into tumor tissue (Bordat et al. 2022). Preclinical studies show that SC administered Ptx–PAAm achieves a bioavailability of 84% and superior antitumor efficacy compared to IV Taxol. Following systemic absorption, the Ptx–PAAm prodrug releases PTX upon linker cleavage, with ∼40% released at 24 h. Rational optimization of the prodrug structure results in significantly decreased C_max_, a key determinant of systemic toxicity, while maintaining sustained drug exposure for 72 h in tumor bearing mice. Ptx–PAAm increases the MTD threefold and expands the therapeutic index through SC administration of this formulation compared to IV administered Taxol (Bordat et al. 2022) (Table 6).

### Paclitaxel-hyaluronidase hydrogel depot

For localized hydrogel depots, one strategy to overcome SC volume limitations is the incorporation of enzymes such as recombinant human hyaluronidase. This enzyme transiently degrades hyaluronan within the SC extracellular matrix, thereby facilitating rapid dispersion of larger injection volumes. This mechanism has been critical to the successful SC formulations such as Herceptin Hylecta and Phesgo (Locke et al. 2019). A SC formulation of PTX combined with hyaluronidase may be feasible, provided that the intrinsic solubility limitations of PTX are adequately addressed. Lee et al. synthesized biodegradable methoxy poly(ethylene glycol) -poly(ε-caprolactone) copolymer (mPEG-PCL) solutions containing PTX, showing temperature-dependent sol-to-gel transitions (Lee et al. 2010). Following SC peritumoral injection, the in situ-forming gel enables localized delivery of PTX to adjacent tumor tissue. The resulting intratumoral depot provides sustained drug release and more effectively inhibits tumor growth over 10 days compared to IV Taxol in mice bearing B16F10 melanoma (Lee et al. 2010) (Table 6).

### Paclitaxel-loaded nanocapsules–thermosensitive gel

PTX/niclosamide nanocrystals incorporated into a poly lactic-co-glycolic acid (PLGA)-PEG-PLGA thermosensitive hydrogel (PN-NCs–Ts gel) constitute an in situ depot system that regulates local and systemic exposure through controlling release kinetics (Zhao et al. 2021). The dual-drug nanocrystals (~ 180 nm) deliver high local payloads at a molar ratio of 1:2. The PN-NCs–Ts gel exhibits slower PTX release (i.e., 53% over 8 days). This sustained release effectively reduces the instantaneous free drug concentration, mitigating C_max_-driven systemic exposure while prolonging intratumoral drug residence. The controlled release kinetics translate into significant efficacy and safety in a TNBC xenograft model. A single intratumoral administration (PTX 20 mg/kg, niclosamide 15 mg/kg) achieves 68.8% tumor growth inhibition and reduces the Ki-67-positive cell fraction to 9.4% compared to 33.6% in saline controls. The formulation also exhibits minimal systemic toxicity, as evidenced by stable body weights and absence of major organ damage compared with Taxol. IV Taxol showed severe allergic reactions with 3/6 deaths, ~ 15% body-weight loss, and increased spleen damage (Zhao et al. 2021). The use of biodegradable excipients also contributed to the minimal toxicity (Nkanga et al. 2020). Consistent with Table 6, these findings indicate that expanding the therapeutic index in depot delivery depends primarily on optimizing local retention and controlled drug exposure, rather than solely on solvent removal or prolonged systemic circulation.

### Paclitaxel nanocrystals gel

PTX nanocrystals (~ 180 nm) dispersed in carbomer 974/poloxamer 407 gel (PTX-NCs-gel) undergo gelation at 33.1 °C, forming an in situ drug reservoir. In a 4T1-luc post-surgery recurrence model, a depot-type therapy was achieved by applying a PTX-NCs-gel (20 mg/kg) directly to the resection bed (Fan et al. 2022). PTX-NCs-gel achieves complete local control with a 0% recurrence rate and fully inhibits lung metastases. These findings indicate that effective suppression of local tumor regrowth directly limits downstream metastatic progression.

The safety profiles highlight the contrast between systemic and locally confined exposure. IV Taxol induces severe systemic toxicity, resulting in a 50% mortality rate due to acute hypersensitivity, ~ 15% body-weight loss, and a ~ fourfold increase in the spleen index. Conversely, the depot-based PTX-NCs-gel completely eliminates these systemic toxicities while preserving strong antitumor and antimetastatic efficacy (Fan et al. 2022). Consistent with Table 6, these findings indicate that expanding the therapeutic index in local delivery depends on optimizing intratumoral retention to minimize C_max_-driven systemic exposure. By localizing drug exposure to the resection margin and avoiding high systemic peaks, the PTX-NCs-gel system effectively balances intratumoral retention with safety constraints, supporting a meaningful expansion of the therapeutic index in localized delivery strategies.

A key unresolved question for SC PTX platforms is their translational feasibility beyond rodent models. Even if systemic tolerability improves, clinical success will depend on injection-site safety in humans, manageable injection volumes, and scalable manufacturing—especially for depot-forming systems, where prolonged local residence may increase the risk of inflammation or fibrosis. Therefore, future studies should prioritize not only efficacy but also standardized local tolerability assessments and clinically relevant administration parameters (Peloso et al. 2021).

## Biological barriers for oral administration of paclitaxel

### P-gp-and CYP3A-mediated first-pass effect of paclitaxel

Functioning as a biological gatekeeper, P-gp recognizes diverse substrates and actively transports them back into the gastrointestinal lumen, thereby limiting their absorption into systemic circulation (Yendapalli et al. 2025). PTX is a high-affinity P-gp substrate; thus, P-gp-mediated efflux is a major determinant of its low oral bioavailability, rendering inhibition or circumvention of this transport a key strategy for developing oral formulations (Srivalli et al. 2012).

PTX undergoes hepatic metabolism to 6α-hydroxy PTX via cytochrome P450 isoform CYP2C8 and to 3’-p-hydroxy PTX via CYP3A4. Evaluation of the relative contributions of these metabolic pathways using human liver microsomes reveals that CYP2C8 represents the predominant pathway for overall drug clearance (Taniguchi et al. 2005). In patients with ovarian cancer, carriers of the CYP2C8*3 variant, which reduces enzyme activity, exhibit an 11% lower clearance of unbound PTX than that of noncarriers. This reduction leads to increased systemic exposure (Bergmann et al. 2011). Clinical case reports further support this association, demonstrating that potent CYP2C8 inhibition by a co-administered clopidogrel metabolite is associated with markedly reduced PTX clearance and an increased risk of peripheral neuropathy (Bergmann et al. 2011).

However, CYP3A4 is the predominant intestinal CYP isoform, accounting for up to 80% of total intestinal CYP content (Watkins 1997). While CYP2C8 is also expressed in the intestine, it is significantly less abundant than CYP3A4 and contributes minimally to overall CYP activity (Grangeon et al. 2021). Therefore, CYP3A4 remains a critical clinical target, particularly for oral formulations. A 2020 Phase I trial evaluated two oral PTX formulations (20 mg, administered twice daily), ModraPac001 and ModraPac005, co-administered with ritonavir (100 mg, administered twice daily), a potent CYP3A4 inhibitor. At this dose, the regimen is feasible and well tolerated, achieving a maximum plasma concentration of 34.6 ng/mL and an AUC of 255 ng·h/mL, approximately half of the evaluable patients showing stable disease (de Weger et al. 2021).

### Absorption site of paclitaxel in the intestine

**Fig. 3:**
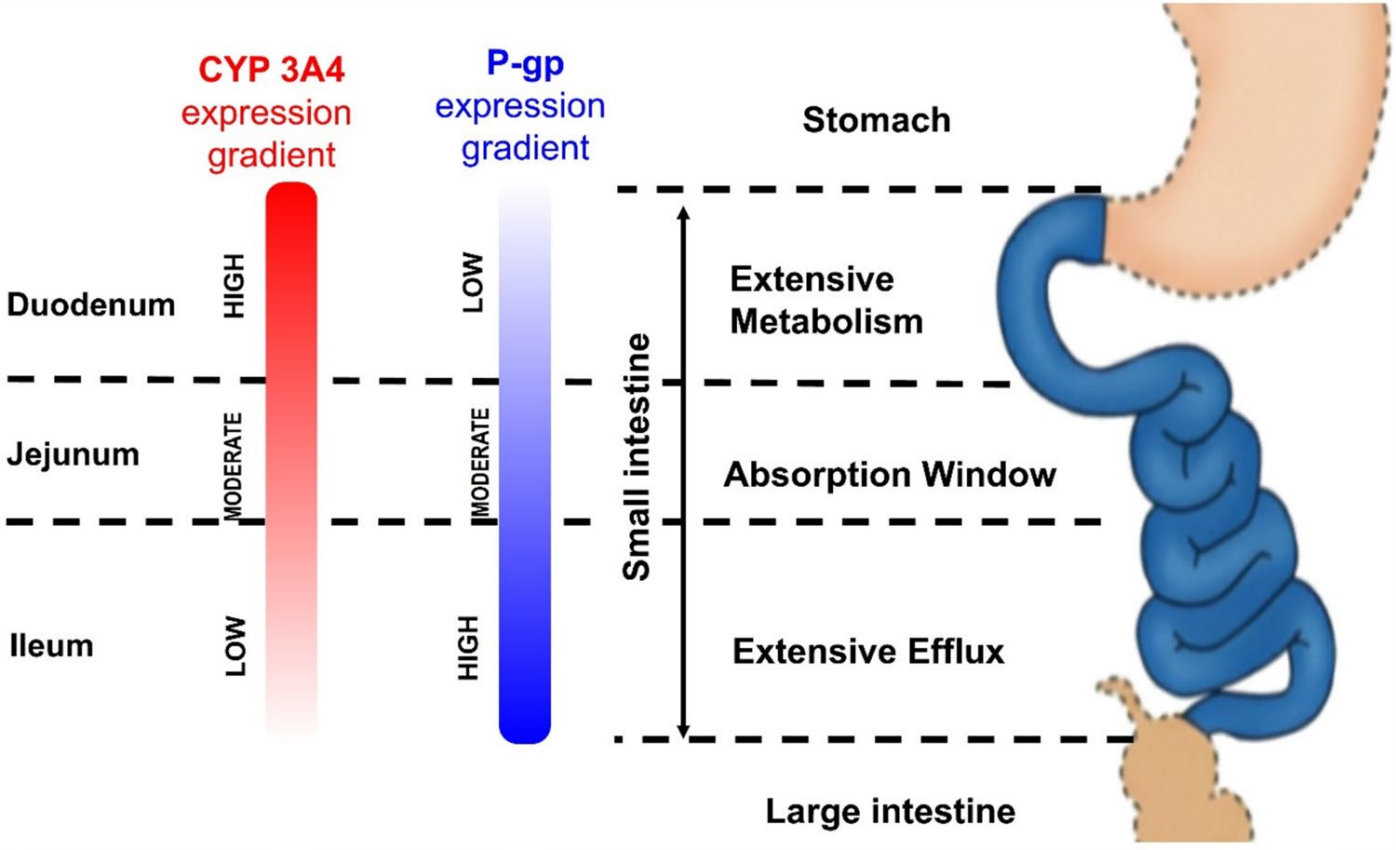
Regional distribution of intestinal CYP3A4 activity and P-gp expression. CYP3A4 activity decreases from the duodenum to the ileum, while P-gp expression increases toward the distal intestine. This inverse gradient highlights the jejunum as the primary site of presystemic metabolism and efflux for orally administered paclitaxel, limiting its bioavailability. CYP3A4, cytochrome P450 3A4; P-gp, P-glycoprotein

The site-specific intestinal absorption of orally administered PTX is determined based on the segment-specific microenvironment, particularly the differential expression of P-gp and CYP3A. Within the small intestine, P-gp expression progressively increases from the proximal jejunum to the distal ileum. This expression gradient results in increased PTX efflux in the distal small intestine (Mai et al. 2021). Conversely, intestinal CYP3A expression is highest in the proximal small intestine and progressively decreases towards the ileum. Consequently, presystemic metabolic elimination of PTX is more pronounced in the proximal region (Watkins 1997; Fig. 3). An effective oral formulation must therefore overcome the P-gp and CYP3A barriers simultaneously or bypass this region entirely (Wilson et al. 2019).

The expression patterns of P-gp and CYP3A suggest that the jejunum provides a comparatively favorable site for PTX absorption. For instance, in a rat in situ closed-loop model, PTX incorporated into galactosamine-grafted micelles shows significantly increased absorption in the jejunum and colon, supporting site-dependent uptake dependent on the formulation (Yang et al. 2015). Furthermore, PTX incorporated into PEGylated nanoparticles shows a 3- to sevenfold increase in the jejunum permeability compared to that of a commercial formulation (Zabaleta et al. 2012). Recent studies also report that oral formulations containing targeting ligands, such as transferrin, enhance overall absorption by promoting uptake through apical enterocyte receptors, particularly in the jejunum (Han et al. 2020).

## Formulation strategy V: overcoming biological barriers for oral administration of paclitaxel

### Co-administration with P-gp inhibitors

The most direct strategy to overcome P-gp-mediated efflux is co-administering a therapeutic agent with a P-gp modulator. Valspodar (PSC-833)—a non-immunosuppressive cyclosporine analog—has been identified as a potent inhibitor of P-gp and CYP3A4. In a Phase I clinical trial, co-administering valspodar (5 mg/kg, per oral) with IV PTX produces a strong pharmacokinetic interaction: the terminal half-life of PTX doubled (8–16 h), its mean residence time increased by 156% (4–11 h), and the formation of 6α-hydroxy PTX increased by 222%. As a result, the MTD decreases to 70 mg/m^2^ (60% of the standard 150 mg/m^2^) to prevent grade 4 neutropenia and ataxia. Despite this adjustment, patients continued to experience DLT, including myelosuppression, ataxia, and hyperbilirubinemia, reflecting excessive PTX accumulation from simultaneous inhibition of metabolic and efflux pathways. Such concurrent inhibition of P-gp and CYP3A4 prolongs PTX exposure and exacerbates systemic toxicity, limiting the clinical utility of first-generation P-gp modulators (ten Tije et al. 2003; Fracasso et al. 2023). However, valspodar has not been evaluated in combination with oral PTX. Therefore, the combined strategy of inhibiting P-gp-mediated efflux and CYP3A4-mediated first-pass metabolism with valspodar was not pursued further, as nonspecific systemic inhibition of both pathways is clinically prohibitive (Darby et al. 2011; Thomas et al. 2017). Consequently, later efforts to develop oral PTX abandoned first- and second-generation inhibitors, including valspodar, and focused instead on gut-specific third-generation P-gp inhibitors or novel formulation-based strategies.

Elacridar (GF120918), tariquidar (XR9576), and zosuquidar (LY335979) are highly potent and selective P-gp inhibitors with minimal off-target effects. In wild-type mice, co-administering elacridar increases PTX oral bioavailability from 8.5–40.2%, yielding pharmacokinetics comparable to those observed in P-gp knockout mice (Malingré et al. 2001). In CYP3A4-humanized mice, elacridar alone increases PTX plasma concentrations by 10.7-fold (Hendrikx et al. 2014). Hubensack et al. report that preadministration of tariquidar increases brain-to-plasma ratios by 15-fold higher than that of valspodar (Hubensack et al. 2008). Similar to earlier P-gp inhibitors, these agents fail to translate robust preclinical potency into meaningful clinical benefit. Phase II and III trials evaluating their combination with chemotherapeutic agents, such as PTX, consistently yield disappointing results, demonstrating acceptable but unremarkable safety profiles and no significant improvement in efficacy outcomes, including objective response rate and survival. For example, a Phase II trial shows that adding tariquidar to taxane- or anthracycline-based therapy in patients with refractory breast cancer results in an ORR of only 6% (Pusztai et al. 2005).

The repeated failure of three generations of systemic P-gp inhibitors highlights a fundamental limitation of this therapeutic strategy. Achieving concentrations sufficient to inhibit P-gp in target tissues, including the gastrointestinal tract and tumors, without inducing unacceptable toxicity proves clinically unattainable (Chung et al. 2016; Lai et al. 2020). This recognition prompts a shift away from systemic modulation toward more targeted approaches.

### Combination with gut specific P-gp inhibitors

**Fig. 4:**
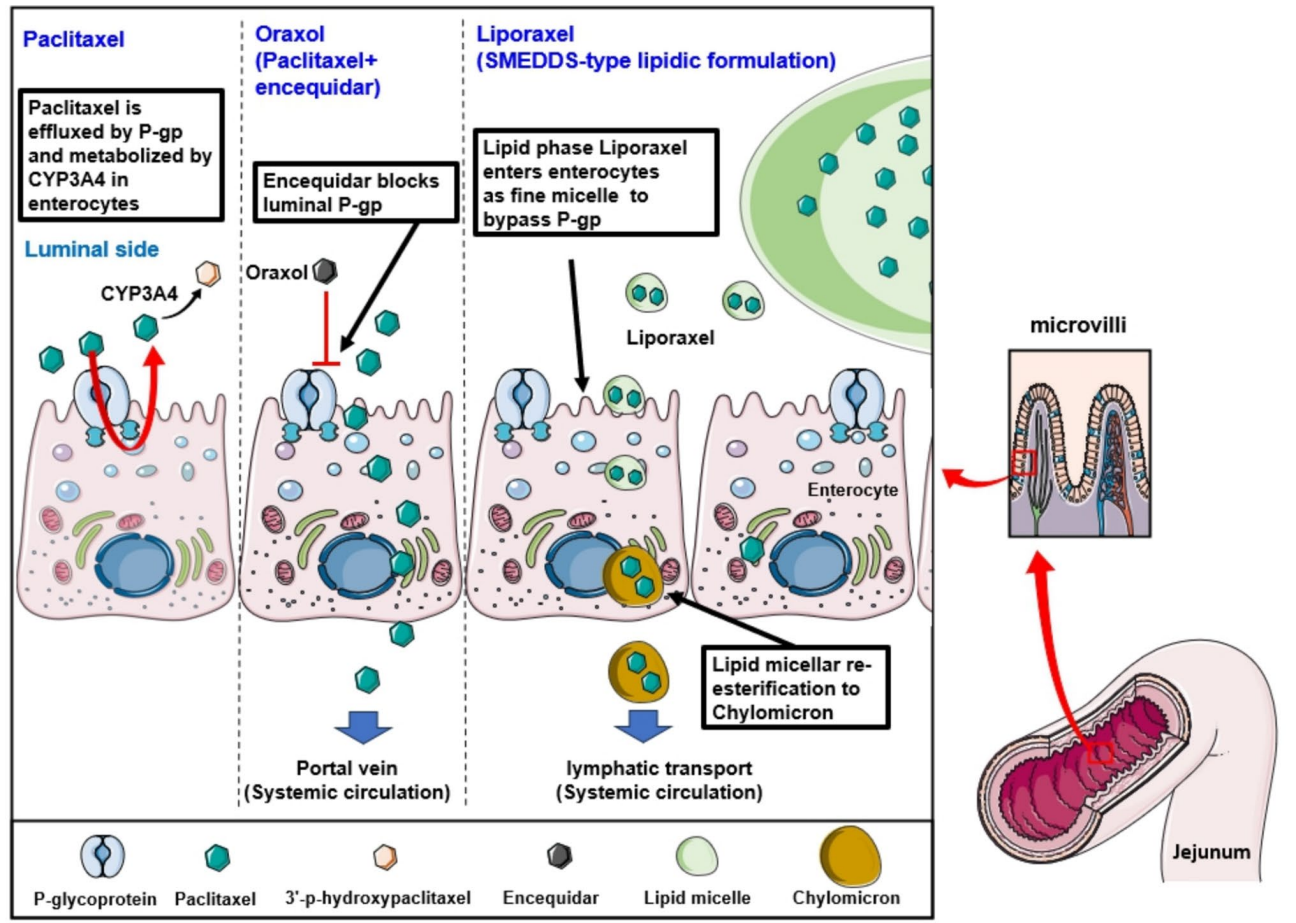
Distinct intestinal absorption mechanisms of paclitaxel formulations. Schematic representation showing absorption pathways for oral paclitaxel formulations in the small intestine. Neat paclitaxel undergoes P-gp-mediated efflux and CYP3A4 metabolism in enterocytes, leading to poor absorption. Oraxol employs the gut-specific P-gp inhibitor encequidar to block luminal efflux and enhance absorption via the portal vein. Liporaxel is delivered as fine lipid micelles, re-esterified into chylomicrons, and transported via the lymphatic system, bypassing intestinal P-gp and first-pass metabolism. P-gp, P-glycoprotein; CYP3A4, cytochrome P450 3A4

The breakthrough in oral PTX development is attributed to a reevaluation of gut-specific P-gp inhibition. This strategy shifts toward a more attainable objective—targeting first-pass efflux at the intestinal epithelium. This approach leads to the development of encequidar (HM30181A), a potent P-gp inhibitor designed to remain minimally absorbed in the gastrointestinal tract (Ma et al. 2022). By selectively inhibiting intestinal P-gp, encequidar enhances PTX absorption while minimizing systemic exposure and the associated toxicities that undermined earlier inhibitors (Janson et al. 2003; Dai et al. 2023) (Fig. 4).

Combining oral PTX with encequidar—marketed as Oraxol—constitutes a CrEL-free, dual-component oral formulation comprising PTX capsules (270 mg/m^2^) co-administered with encequidar tablets (15 mg). This strategy demonstrates substantial clinical benefits: Oraxol achieves an oral PTX bioavailability of 40–45% relative to that of the intravenous formulation, maintains comparable systemic exposure, and significantly reduces solvent-related hypersensitivity reactions. In metastatic breast cancer, Oraxol achieves an ORR of 36% and mPFS of 8.4 months, thereby confirming its clinical feasibility (Ma et al. 2022; Dai et al. 2023).

A randomized crossover pharmacokinetic study directly compared the oral formulation with standard IV PTX, demonstrating that the oral regimen achieves systemic exposure comparable to that of an 80 mg/m^2^ intravenous dose, with a mean absolute bioavailability of 12% (Jackson et al. 2021). Definitive clinical validation is provided by a pivotal Phase III trial in metastatic breast cancer. The trial reports that oral PTX in combination with encequidar is superior to conventional intravenous PTX with respect to the primary endpoint, achieving a higher confirmed radiographic response rate (36% vs. 23%; p = 0.01). The study also demonstrates favorable trends toward improved mPFS (8.4 months vs. 7.4 months) and mOS (22.7 months vs. 16.5 months) (Rugo et al. 2023).

However, its clinical development was discontinued following the early termination of three Phase III trials, including one showing a higher incidence of adverse events when combined with the chemotherapeutic agent vinorelbine (Modok et al. 2006). Despite receiving marketing approval in South Korea, Oraxol (PTX 270 mg/m^2^ + encequidar 15 mg) has not obtained regulatory approval in major Western markets, including the United States and the United Kingdom (Yu et al. 2025). While Oraxol demonstrates promising clinical efficacy—particularly in improving PFS in patients with triple-negative breast cancer—it shows reduced neuropathy but increased incidence of serious neutropenic infections (Yu et al. 2025; Zhang et al. 2025a, b). This highlights a safety trade-off potentially necessitating more precise risk stratification and confirmatory survival data to establish a robust benefit–risk profile (Rugo et al. 2023). Recently, the FDA issued a Complete Response Letter regarding this combination in metastatic breast cancer, indicating that improvements in response rates do not necessarily outweigh residual uncertainties concerning safety and long-term efficacy (Wang et al. 2022).

### Prolonged gastrointestinal residence time in jejunum

The intrinsic peristaltic motility of the gastrointestinal tract propels its contents forward, thereby limiting the residence time of orally administered drugs. Rapid gastrointestinal transit is particularly detrimental for poorly soluble compounds, which require additional time to dissolve and be absorbed (Lonkar et al. 2025). Consequently, future formulations should be designed to prolong gastric residence time and decouple drug delivery from physiological transit. This can be achieved through mucoadhesion (adhering to the gastric mucosa), expansion (swelling to a size that prevents passage through the pylorus), and flotation (maintaining buoyancy on gastric fluids by incorporating low-density materials) (Zaid et al. 2024).

Preclinical formulation research aimed at improving oral PTX absorption has increasingly focused on the jejunum as the preferred absorption site, considering the differential CYP3A4 and P-gp expression pattern. The jejunal segment serves as an “optimal absorption window,” strategically positioned to minimize extensive CYP3A4-mediated presystemic metabolism in the proximal intestine and reduce P-gp-mediated drug efflux in the distal intestine (Fig. 3). For example, a study evaluating PTX-loaded glycyrrhizic acid micelles attributed the observed increase in oral bioavailability primarily to enhanced absorption in the jejunum and colon (Yang et al. 2015). Furthermore, multiple formulation strategies are designed to leverage these regional adsorption advantages. PTX-loaded nanoparticles formulated from hydrophobically modified glycol chitosan exhibited pronounced mucoadhesive properties, resulting in enhanced local drug delivery and prolonged retention in the excised rat ileum (Soundararajan et al. 2016). In addition, folate-engineered zein nanoparticles targeting intestinal epithelial folate receptors extended gastrointestinal residence in rats for approximately 24 h. This approach produced a > sevenfold increase in PTX plasma concentrations in rabbits compared to that of the unformulated drug (Somaida et al. 2025). These findings are consistent with decreasing proximal-to-distal gradient of intestinal CYP3A4 expression and the corresponding increase in P-gp activity along the small intestine. Collectively, these anatomical and physiological patterns reinforce the rationale for jejunum-targeted formulation design as a central strategy to address the low oral bioavailability of PTX. A principal limitation of the fixed “jejunal window” paradigm is inherent biological variability. Intestinal expression levels of CYP3A4 and drug transporters vary substantially between individuals, and disease states or concomitant medications may further alter gastrointestinal transit time and metabolic activity. These factors may increase interindividual variability in oral drug exposure, even for well-designed formulations. Accordingly, jejunum-targeted strategies should ideally be integrated with approaches that actively mitigate such variability, including food-effect control, drug-drug interaction management, and model-informed dose optimization (Effinger et al. 2019; Vinarov et al. 2021).

### P-gp bypassing formulation

Liporaxel (DHP107) is an orally administered PTX formulation developed using the patented DH-LASED lipid-based technology of Daehwa Pharmaceutical (Seoul, Republic of Korea). Liporaxel contains monoolein (55.0%), tricaprylin (27.5%), and polysorbate 80 (16.5%) (Jang et al. 2017; Chae et al. 2024). These excipients solubilize PTX into a microemulsion that spontaneously forms nanosized lipid droplets (~ 50–150 nm) upon exposure to gastrointestinal fluids. The droplets are subsequently re-esterified into chylomicrons and transported via the intestinal lymphatic pathway. Upon hydration, the formulation transitions into a lipidic sponge phase that adheres to the intestinal villi for approximately 24 h, thereby prolonging jejunal residence time, enhancing chylomicron-mediated lymphatic uptake, and maximizing the effective absorption window for PTX while minimizing P-gp-mediated efflux (Hong et al. 2007; Wu et al. 2014; Dokania et al. 2015; Jang et al. 2018; Moon et al. 2021; Yousef et al. 2024) (Fig. 4). This regional absorption advantage is well established. In a closed-loop intestinal absorption study conducted in rats, the lipid-based oral PTX formulation Liporaxel shows a significantly higher AUC in the jejunal segment than in the ileum and colon (Jang et al. 2017). In a single-arm Phase II pharmacokinetic study, 13 Caucasian patients with HER2-negative metastatic breast cancer received Liporaxel at 200 mg/m^2^ orally twice daily on days 1, 8, and 15 of a 28-day cycle. The median T_max_ was 2.17 h (range, 1.92–4.08 h), C_max_ was 330 ng/mL with a CV of 31.1%, and AUC was 1233 ng·h/mL with a CV of 30.3%. These pharmacokinetic parameters were consistent with those observed in a Phase I Korean cohort (C_max_ 235 ng/mL with CV of 43.9%; AUC 1348 ng·h/mL with CV of 19.7%), indicating no clinically relevant pharmacokinetic differences between Asian and Caucasian patients (Rugo et al. 2021). Despite these mechanistic advantages, oral lipid-based platforms have exhibited exposure variability and nonlinear pharmacokinetics at higher dose levels. For DHP107 specifically, the absence of dose-proportional exposure beyond certain dose thresholds, along with potential food-related effects on intestinal adhesion or transporter induction, has been suggested as a contributor to pharmacokinetic variability. These considerations underscore the need for rigorous characterization of food effects and schedule optimization when positioning DHP107 against established intravenous regimens (Jang et al. 2017).

Liporaxel (200 mg/m^2^ administered orally twice daily on days 1, 8, and 15 of a 28-day cycle) was initially approved in South Korea in 2016 for the treatment of advanced gastric cancer and subsequently received approval from the NMPA of China in 2024 (Kim et al. 2021) (Table 1). The formulation is currently being evaluated in global clinical trials, including the FDA-regulated OPERA Phase II study in the US, EMA-monitored OPTIMAL Phase III trial in Europe, and NMPA-reviewed OPTIMAL trial in China (Kim et al. 2020; Pluard et al. 2020). Preliminary results from the NMPA-reviewed Chinese cohort indicate favorable PFS outcomes, supporting its potential as a first-in-class oral therapy (Kim et al. 2020). In these studies, Liporaxel demonstrates efficacy comparable to that of IV PTX while significantly reducing the incidence of hypersensitivity reactions and peripheral neuropathy—benefits attributed to its oral administration of lipid-based SMEDDS design (Kim et al. 2021).

In the global Phase III OPTIMAL trial, 549 patients with hormone receptor–positive (HR +) or triple-negative/HER2-negative metastatic breast cancer—who had received at least one prior line of endocrine therapy but no chemotherapy for metastatic disease—were randomized to receive oral Liporaxel (200 mg/m^2^ administered twice daily on days 1, 8, and 15 of a 28-day cycle) or IV Taxol (80 mg/m^2^ administered weekly). At a median follow-up of 38.8 months, Liporaxel demonstrated non-inferiority to IV PTX for the primary endpoint of PFS (mPFS: 10.02 months vs. 8.54 months; mOS: 32.95 months vs. 32.46 months). In the HR + /HER2– subgroup, clinical outcomes favored Liporaxel, with longer mPFS (10.74 months vs. 9.07 months) and a higher ORR (45.8% vs. 39.7%) than that of the IV Taxol. Liporaxel was associated with lower incidences of peripheral neuropathy (37.9% vs. 48.3%), hypersensitivity reactions, and infusion-related events, but higher rates of grade ≥ 3/4 neutropenia (67.2% vs. 29.7%) and febrile neutropenia (6.14% vs. 0.76%). Discontinuation owing to adverse events was comparable between the two treatment arms (12.3% vs. 8.8%; p = 0.208) (Kang et al. 2018). The study demonstrates non-inferior efficacy and manageable tolerability compared to that of IV PTX, suggesting that Liporaxel may represent a more convenient and effective treatment option for patients with HER2-negative metastatic breast cancer.

## Future perspectives

**Fig. 5:**
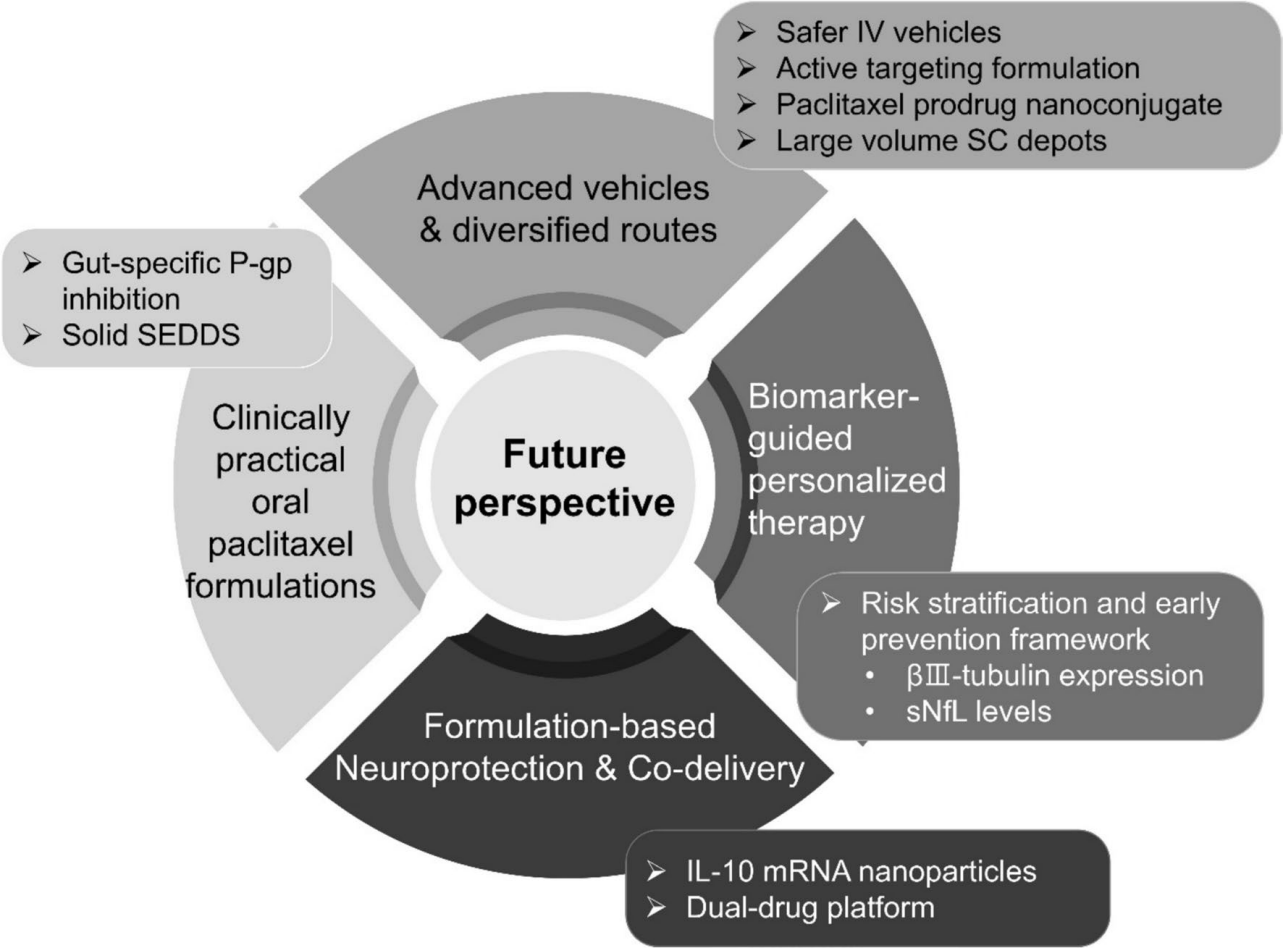
Future perspectives in paclitaxel therapy

Based on recent advances in PTX formulation, Fig. 5 summarizes the future perspectives of next-generation PTX therapy. Meaningful advances in PTX formulation are expected at the intersection of four key domains: (i) innovative PTX formulations to reduce intrinsic PTX toxicities while maintaining the efficacy; (ii) expansion of administration routes, including oral systems designed to modulate intestinal P-gp/CYP3A barriers; (iii) practical SC depots for loco-regional delivery; and (iv) integration of predictive biomarkers, including βIII-tubulin and sNfL, to support risk stratification and early prevention of neuropathy (Fig. 5).

### Innovative paclitaxel formulations to reduce intrinsic toxicities while maintaining the efficacy

The clinical success of Abraxane and other lipospmal and micellar formulations (Table 2 and 3) demonstrates the viability of CrEL–free IV formulations; however, peripheral neuropathy and myelosuppression remain DLTs (Qutub et al. 2025). Ongoing formulations under clinical evaluation focus on optimizing particle size, surface chemistry, and lipid composition to increase the plasma stability and distribute to tumor tissue via EPR effect and to reduce peripheral nerve and bone marrow exposure, as suggested by preclinical liposomal systems that limit PTX accumulation in neuronal tissues. While these platforms improve tolerability by reducing systemic toxicity and increasing the maximum tolerated concentration, they often fail to boost tumor-site efficacy, which limits the effective dose. This suggests that relying solely on passive EPR-mediated accumulation is insufficient for meaningful therapeutic expansion. According to Lu and Prakash (2021), future strategies must mechanistically overcome barriers to tumor access and intratumoral transport. Specifically, innovative PTX formulations—such as the self-assembled PTX prodrug nanoparticles detailed in Table 5—that actively exploit microenvironment modulation or transcytosis pathways warrant clinical evaluation to achieve superior therapeutic outcomes with manageable toxicity.

### Expansion of administration routes to clinically practical oral formulations

Future strategies should prioritize site-specific approaches over non-selective inhibition (Hua 2020). Formulations may target the jejunum to exploit a favorable transporter–enzyme balance using pH-dependent or time-controlled release systems. Then, the use of minimally absorbed, intestine-specific P-gp inhibitor with that facilitates clinically relevant oral PTX exposure is critical. Recent studies confirm that gut-localized P-gp inhibition represents a well-established and generalizable approach for oral PTX development, as supported by pharmacokinetic studies of oral docetaxel/encequidar combinations (Smolinski et al. 2021; Rugo et al. 2023). Beyond encequidar, a new generation of intestine-specific P-gp inhibitors is emerging*.* Compound 16c, a benzofuran-fused piperidine derivative, is a highly potent, gut-restricted P-gp inhibitor essentially non-absorbed (F ~ 0.8%) but enhances oral PTX exposure more effectively than encequidar, achieving high therapeutic efficacy with low systemic toxicity in an MDA-MB-231 xenograft model (Zhou et al. 2024).

Second, lipid-based formulations that enhance lymphatic transport may reduce reliance on P-gp-mediated efflux and partially bypass hepatic first-pass metabolism is also emerging for oral PTX formulation (Zhang et al. 2021). Recent studies have extended this concept from liquid SEDDS to solid SEDDS. For example, cabazitaxel solid SEDDS achieved approximately tenfold higher absolute oral bioavailability than a simple cabazitaxel solution in rats (17.3% vs. 1.7%) while maintaining favorable flowability, tabletability, and re-emulsification properties after solidification (Sun et al. 2024). While PTX has not been translated as a clinically advanced solid SEDDS, recent reviews recognize solidified SEDDS as a versatile platform for highly lipophilic drugs. These findings reveal that Liporaxel-like oral PTX formulations could ultimately be developed as capsule- or tablet-based solid SEDDS products (Baek and Jin 2025; Uttreja et al. 2025).

### Expansion of administration routes to subcutaneous depot

Recent advances in large-volume SC delivery and on-body drug delivery systems for monoclonal antibodies indicate a potential next step for taxanes beyond syringe-based administrations (Sunstrom and Sunstrum 2025; Wu et al. 2025). Wearable or patch-based injectors are now clinically employed to administer 5–10 mL of viscous biologic formulations in home or outpatient settings, demonstrating comparable safety and clear improvements in patient and healthcare provider workflow (Green et al. 2024).

The feasibility of subcutaneous (SC) administration hinges on developing high-concentration, precipitation-resistant formulations. These must deliver therapeutic doses within a 2–5 mL volume for manual injections, or up to 10–20 mL when facilitated by delivery devices or hyaluronidase-mediated dispersion (Kang et al. 2026). Furthermore, preclinical models suggest that injectable in situ-forming hydrogels—which create a localized drug depot—can provide more sustained tumor control and reduced systemic toxicity (Wang et al. 2023). Consistent with this finding, PTX prodrug nanocrystal-loaded in situ depot system containing hyaluronidase seems to be more plausible considering injection-site safety in humans, manageable injection volumes, and scalable manufacturing. This system can provide prolonged systemic PTX exposure while minimizing systemic and injection site toxicities.

### Biomarker-guided personalized therapy to support risk stratification and early prevention of neuropathy

βIII-tubulin remains one of the most advanced candidate biomarkers for taxane-related efficacy and resistance. High βIII-tubulin expression is consistently associated with poor OS in NSCLC, a relationship that appears largely independent of prior taxane exposure. In contrast, in breast and ovarian cancers, the predictive value of βIII-tubulin is more context-dependent, varying with the specific regimen and line of therapy. In tumors exhibiting high βIII-tubulin expression, ixabepilone is being reconsidered as a treatment option for patients unlikely to benefit from further taxane therapy (Dumontet et al. 2009; Rau et al. 2020). Concurrently, sNfL emerges as a sensitive and early biomarker of axonal injury and chemotherapy-induced peripheral neuropathy. Prospective studies and expert reviews indicate that increasing sNfL levels may help predict clinically relevant neurotoxicity and guide preemptive dose modification during PTX therapy (Andersen et al. 2024).

Collectively, βIII-tubulin (as an efficacy and resistance biomarker) and sNfL (neurotoxicity biomarker) constitute a practical dual-biomarker framework. Within this framework, decisions regarding continuing PTX therapy, transition to non-taxane or epothilone-based regimens, and modification of dose intensity or treatment schedule may become increasingly quantitative and individualized.

An emerging direction involves the development of systems designed to actively counteract PTX-induced neurotoxicity while preserving antitumor efficacy. Recent preclinical strategies highlight this paradigm shift from “improved delivery” to the “co-delivery of neuroprotective agents.” For example, via mater–penetrating lipopolymer nanoparticles engineered to deliver IL-10 mRNA have been developed to selectively target glial cells within the central nervous system. This strategy attenuates PTX-induced peripheral neuropathy without reducing systemic PTX exposure (Lin et al. 2025). Similarly, topical capsaicin nanocrystal gels and small-molecule mitophagy inducers, including ALT001, alleviate taxane-related neuropathy in rodent models, functioning adjunctively rather than as substitutes for the anticancer agent (Im et al. 2024; Hade et al. 2025). Furthermore, hydrogel–nanoparticle dual-drug platforms offer a conceptual framework for the development of future PTX depots. These systems are capable of co-encapsulating the cytotoxic agent and a neuroprotective or anti-inflammatory payload within a single injectable formulation. This design facilitates sustained peritumoral release of PTX while concurrently delivering protective signals to vulnerable neuronal structures (Liu et al. 2024; Yoon et al. 2025). Coupled with objective toxicity measures, such as sNfL, these co-delivery and neuroprotection-focused formulation strategies represent a potential future direction. In this approach, PTX formulation, dosing, and biomarker-based monitoring may be jointly optimized to maintain tumor control while proactively limiting cumulative neurotoxicity.

## Conclusion

The sustained clinical relevance of PTX reflects its highly specific microtubule-targeting pharmacology and the continued advancement of formulation science. In this review, the progression of the field in overcoming three fundamental barriers previously limiting its therapeutic potential was examined. In particular, the CrEL-related constraint was effectively addressed through the development of solvent-free nano-formulations, including Abraxane and micellar and liposomal formulation, thereby reducing hypersensitivity reactions and enabling more predictable, linear pharmacokinetics.

Efforts to address limited oral bioavailability involve co-administration with the gut-specific P-gp inhibitor encequidar, as in Oraxol, and the use of lipid-based SMEDDS, such as Liporaxel, which promote lymphatic uptake and bypass P-gp–mediated efflux. Additionally, intrinsic DLTs prompt the development of nanocarriers designed to modify biodistribution. Innovative PTX formulations that actively exploit microenvironment modulation or transcytosis pathways warrant clinical evaluation to achieve superior therapeutic outcomes with manageable toxicity.

In summary, the next major advancement in PTX therapy is unlikely to involve a new chemical entity. Instead, it is expected to reflect a more sophisticated approach to delivering existing PTX, characterized by flexible administration routes, biomarker-guided treatment decisions, and controlled systemic exposure using active targeting. Success is defined by improvements in survival endpoints and measurable enhancements in patient experience, including shorter infusion times, elimination of routine premedication, and reduced debilitating toxicities. Expanding the therapeutic safety margin requires strategies. The decisive translational determinant is whether a formulation achieves effective tumor concentration by precisely controlling the pharmacokinetic time course of active PTX. Consequently, future development paradigms must prioritize strategies that demonstrate mechanistically justified control of free-drug kinetics and quantifiable tumor-to-DLT tissue exposure ratios, rather than platforms designed to extend plasma circulation.

## Data Availability

This is a review article. All data discussed are from previously published studies, which are cited in the reference list. No new datasets were generated.
